# Strategies by which WWOX-deficient metastatic cancer cells utilize to survive via dodging, compromising, and causing damage to WWOX-positive normal microenvironment

**DOI:** 10.1038/s41420-019-0176-4

**Published:** 2019-05-21

**Authors:** Pei-Yi Chou, Feng-Jie Lai, Yu-An Chen, Yong-Da Sie, Hsiang-Ling Kuo, Wan-Pei Su, Chia-Yun Wu, Tsung-Yun Liu, Kuang-Yu Wen, Li-Jin Hsu, Chun-I Sze, Nan-Shan Chang

**Affiliations:** 1 Institute of Molecular Medicine, National Cheng Kung University, Tainan, Taiwan, ROC; 2 Department of Dermatology, Chi-Mei Medical Center, Tainan, Taiwan, ROC; 3 Advanced Optoelectronic Technology Center, National Cheng Kung University, Tainan, Taiwan, ROC; 4 Department of Medical Laboratory Science and Biotechnology, National Cheng Kung University, Tainan, Taiwan, ROC; 5 Department of Cell Biology and Anatomy, National Cheng Kung University, Tainan, Taiwan, ROC; 6 Department of Neurochemistry, New York State Institute for Basic Research in Developmental Disabilities, Staten Island, NY USA; 7 Graduate Institute of Biomedical Sciences, College of Medicine, China Medical University, Taichung, Taiwan, ROC

## Abstract

Proapoptotic tumor suppressor WWOX is upregulated in the early stage of cancer initiation, which probably provides limitation to cancer growth and progression. Later, WWOX protein is reduced to enhance cancer cell growth, migration, invasiveness and metastasis. To understand how WWOX works in controlling cancer progression, here we demonstrate that apoptotic stress mediated by ectopic WWOX stimulated cancer cells to secrete basic fibroblast growth factor (bFGF) in order to support capillary microtubule formation. This event may occur in the cancer initiation stage. Later, when WWOX loss occurs in cancer cells, hyaluronidase production is then increased in the cancer cells to facilitate metastasis. We determined that inhibition of membrane hyaluronidase Tyr216-phosphorylated Hyal-2 by antibody suppresses cancer growth in vivo. WWOX-negative (WWOX-) cells dodged WWOX+cells in the microenvironment by migrating individually backward to avoid physical contacts and yet significantly upregulating the redox activity of WWOX+parental cells or other WWOX+cell types for causing apoptosis. Upon detecting the presence of WWOX+cells from a distance, WWOX- cells exhibit activation of MIF, Hyal-2, Eph, and Wnt pathways, which converges to MEK/ERK signaling and enables WWOX- cells to evade WWOX+cells. Inhibition of each pathway by antibody or specific chemicals enables WWOX- cells to merge with WWOX+cells. In addition, exogenous TGF-β assists WWOX- cells to migrate collectively forward and merge with WWOX+cells. Metastatic WWOX- cancer cells frequently secrete high levels of TGF-β, which conceivably assists them to merge with WWOX+cells in target organs and secure a new home base in the WWOX+microenvironment. Together, loss of WWOX allows cancer cells to develop strategies to dodge, compromise and even kill WWOX-positive cells in microenvironment.

## Introduction

Proapoptotic tumor suppressor WW domain-containing oxidoreductase, designated WWOX, FOR or WOX1, is known to limit cancer growth and metastasis^1–5^. However, WWOX is even crucial in maintaining physiological settings, rather than functioning in tumor suppression. Null mutations of *WWOX*/*Wwox* gene cause severe neural diseases (e.g., epileptic encephalopathy, microcephaly, and spinocerebellar ataxia), metabolic disorders (including lipid, cholesterol, and glucose metabolism), disorder of sex differentiation, and early death in the newborns^2,6,7^. Spontaneous tumor formation is rarely found in the WWOX-deficient newborns. Importantly, *WWOX* gene is one of the 5 recently discovered risk factors in Alzheimer’s disease^8^. WWOX interacts with specific cytosolic proteins, mainly functioning in normal cell physiology and death^1–5^ and metabolism such as glycolysis, fatty acid degradation and acetyl-CoA generation^9^.

WWOX localizes, in part, in the mitochondria via its *C*-terminal short-chain alcohol dehydrogenase/reductase (SDR) domain^5,10,11^. WWOX maintains the mitochondrial respiratory function^10,11^ and induces mitochondrial apoptosis if overexpressed^10,12,13^. During the early stage of cancer progression, WWOX is Tyr33 phosphorylated (pY33-WWOX) and significantly upregulated, which restricts cancer initiation in vivo^14,15^. Transiently overexpressed pY33-WWOX induces apoptosis in vitro^10,16,17^. Significantly upregulated pY33-WWOX is associated with neuronal death in vivo^18,19^.

During lymphocytic cell differentiation, pY33-WWOX is reduced and switched to S14 phosphorylation^1,20^, which participates in the IκBα/ERK/pS14-WWOX signaling for cell differentiation. Unfortunately, pS14-WWOX is significantly upregulated during the progression of cancer and Alzheimer’s disease^1,2,21,22^. A naturally occurring zinc finger-like peptide Zfra suppresses S14 phosphorylation and consequently inhibits cancer growth^21^ and reduces the symptoms of Alzheimer’s disease^22^.

Loss of WWOX upregulates the JAK2/STAT3 pathway for driving cancer metastasis in triple negative breast cancer cells^23^. Loss of WWOX in ovarian cancer cells enhances migration and metastasis due to altered cell and matrix protein interactions^24^. WWOX suppresses the expression of RUNX2 and blocks the invasion and metastasis of osteosarcoma and lung cancer cells^25,26^. Here, we investigated how cancer cells survive at an early stage under the apoptotic pressure of upregulated pY33-WWOX in vivo^14,15^. How loss of WWOX in cancer cells enhances their migration and metastasis was examined. We determined the behavioral changes of WWOX-deficient (WWOX–) cells in a WWOX-positive (WWOX+) microenvironment.

## Results

### WWOX downregulation induces hyaluronidase production and increases cell migration in vivo

**Fig. 1:**
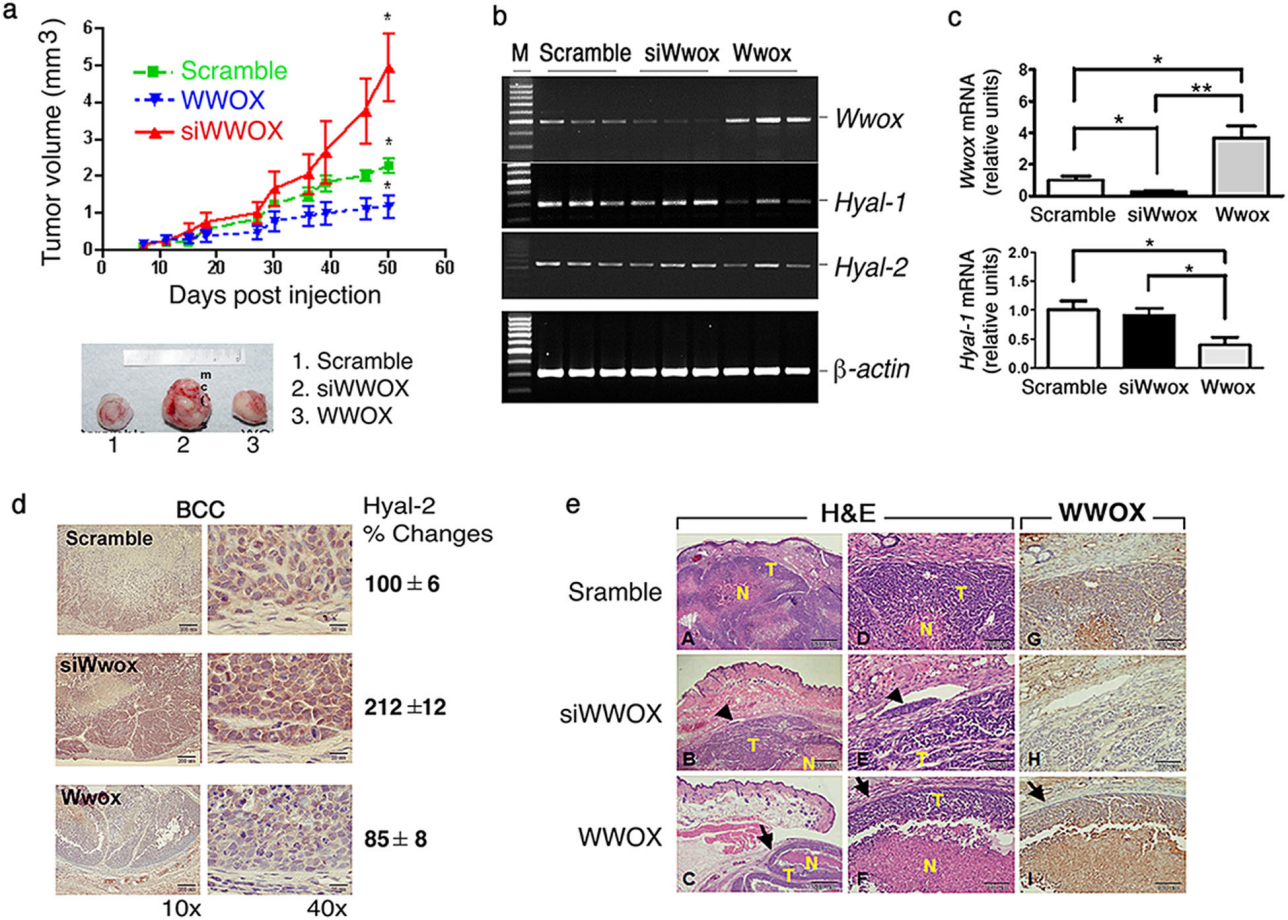
Silencing of WWOX expression induces hyaluronidase expression and increases cancer cell migration in vivo. **a** Stable transfectants of BCC cells expressing a scrambled sequence, siRNA targeting *WWOX*, or ectopic WWOX protein were established. The kinetics of the tumor growth in nude mice was measured. **b, c** By RT-PCR, WWOX-overexpressing BCC cells had more *WWOX* mRNA than cells expressing siWWOX or a scrambled sequence. The mRNA levels of Hyal-1 and Hyal-2 of high WWOX-expressing cells were significantly lower than si*WWOX* tumors. The levels of β-actin mRNA were used as an internal control. Statistical analysis: *p < 0.01, **p < 0.001 (compared with each other group, by one-way ANOVA and Tukey’s Multiple Comparison Test). **d** Mice were sacrificed 50 days post tumor cell injections. In the tumor sections, knockdown of *WWOX* significantly increased the expression of Hyal-2 protein. **e** Lymphatic invasion of WWOX-knockdown BCC cells is shown in representative photomicrographs (see arrowheads; H&E stain). Cyst-like demarcation structure is observed in the tumor nodules from the mice injected with BCC cells overexpressing WWOX (see arrows). By immunohistochemistry, expression of WWOX is also shown. T, tumor cells; N, necrotic areas

High levels of hyaluronidases suppress WWOX expression and increase cancer metastasis^5,10,27,28^. Here, by direct knockdown of WWOX expression in human skin basal cell carcinoma (BCC) using small interfering RNA (si*WWOX*)^16^, BCC tumors grew 4 times larger than those cells expressing ectopic *WWOX*, and 2.5 times larger than the control tumors expressing a scrambled DNA (Fig. 1a).

By quantitative RT/PCR, *HYAL1* and *HYAL2* mRNA transcripts were significantly reduced in WWOX-expressing BCC cells (Fig. 1b). In contrast, *HYAL1* mRNA transcripts were increased in WWOX-knockdown cells (Fig. 1c). BCC cells were grown in nude mice for 50 days. Tumor lesions showed that Hyal-2 was significantly upregulated in the WWOX knockdown BCC cells (Fig. 1d; ~100% increase). BCC-expressing WWOX had a reduced Hyal-2 expression (Fig. 1d; ~15% reduction). WWOX-knockdown cells gained increased migratory activity in vivo, as they were found in the lymphatic vessels (Fig. 1e).

### Suppression of cancer cell growth by Hyal-2 antibody in mice

T/B cell-deficient NOD-SCID mice received Hyal-2 or pY216-Hyal-2 antiserum via tail vein injections once per week for 3 consecutive weeks^21,22^, followed by resting for 2 weeks and then inoculating with mouse melanoma B16F10 cells. Both Hyal-2 antibodies effectively blocked B16F10 growth (Fig. S1a). PBS or normal serum had no effects (Fig. S1a). Similarly, T cell-deficient nude mice received an aliquot of Hyal-2 IgG or normal serum IgG (in 100 μl PBS) in 3 consecutive days, followed by inoculating with B16F10 cells. Hyal-2 IgG inhibited B16F10 growth (Fig. S1b). Hyal-2 antiserum also suppressed BCC growth in nude mice (Fig. S1c).

In survival experiments, BALB/c mice were inoculated with syngeneic breast cancer 4T1 cells in subcutaneous sites of both flanks. When tumors grew up to approximately 150 cubic mm, each control mouse (5 in total) received an aliquot of diluted normal rabbit serum via tail vein injections for 3 times. Experimental mice (5 in total) received diluted rabbit antiserum against pY216-Hyal-2. pY216-Hyal-2 antiserum prolonged the mouse survival (Fig. S2), suggesting that pY216-Hyal-2 supports cancer cell growth in vivo. Hyal-2 is a GPI (Glycosylphosphatidylinositol) anchor-linked membrane protein, which degrades hyaluronan and participates in the Hyal-2/WWOX/Smad4 signaling ^5,29-31^. High levels of hyaluronidases support cancer cell growth^30,31^.

### Apoptotic stress caused by overexpressed WWOX allows BCC cells to secrete bFGF for capillary tube formation

**Fig. 2:**
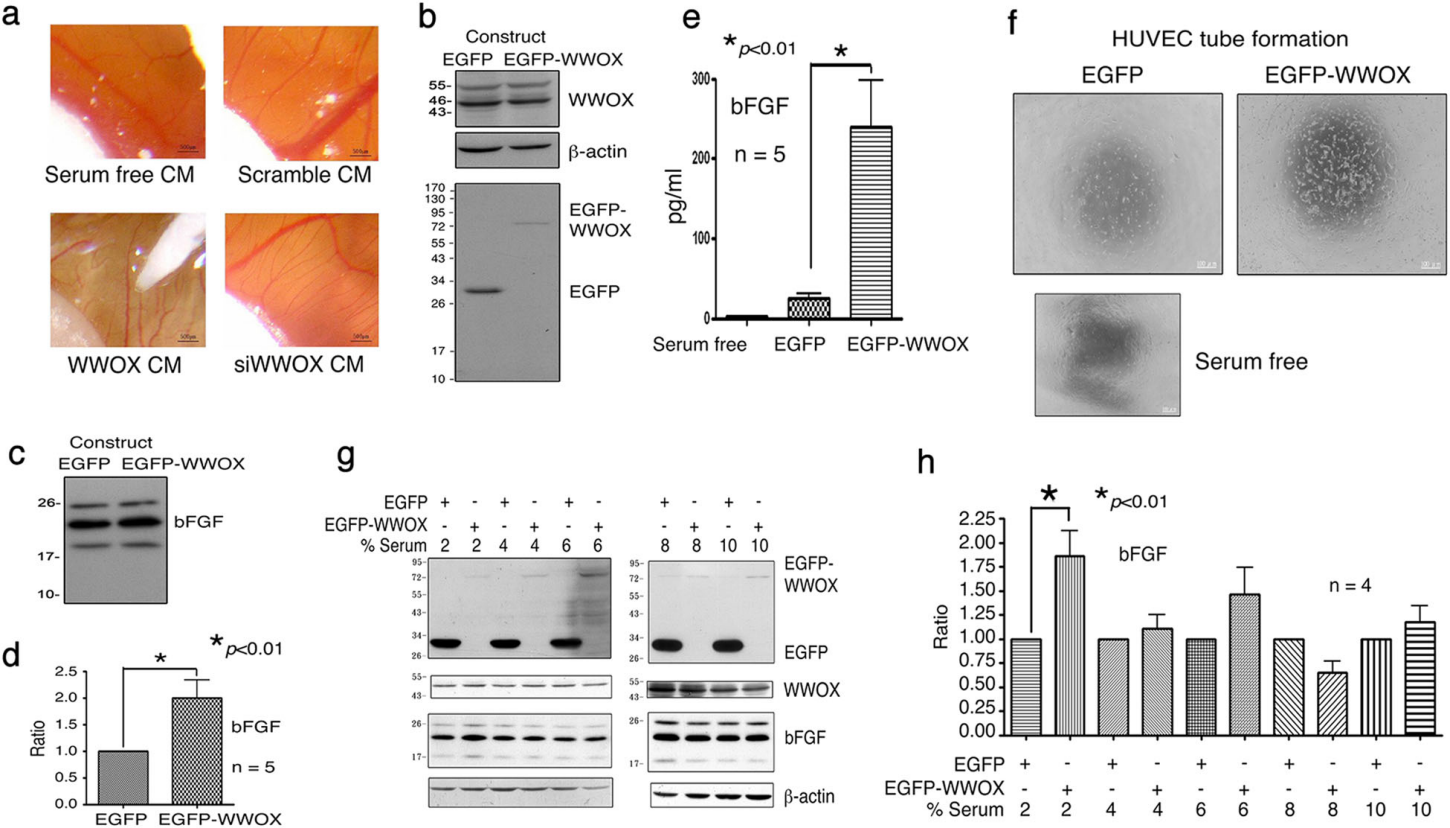
WWOX-overexpressing BCC cells secrete bFGF to support microtubule formation by HUVEC cells. **a** The condition media (CM) from the stable transfectants of BCC cells, as described above, were used to carry out CAM assay using chick embryos. **b** Endogenous WWOX and ectopic EGFP and EGFP-WWOX proteins are shown in the stable transfectants. **c, d** Expression of bFGF protein is shown in the BCC stable transfectants with EGFP and EGFP-WWOX cDNA constructs. The level of bFGF protein in the EGFP-WWOX-expressing BCC is almost double that EGFP cells. **e** EGFP-WWOX-expressing BCC secreted greater amounts of bFGF protein than EGFP cells, as determined by ELISA. **f** The CM from the EGFP-WWOX-expressing BCC supported the tube formation of HUVEC cells. The serum-free medium and the CM form EGFP-expressing BCC had no effect. **g, h** The effect of serum on the production of bFGF is shown in EGFP and EGFP-WWOX-expressing BCC cells

We examined whether BCC cells tackle the overexpressed WWOX-mediated apoptosis^10,16,17^. By chick chorioallantoic membrane (CAM) assay, chick embryos were treated with condition media (CM) from a BCC stable transfectant-expressing EGFP, EGFP-WWOX, or a scrambled sequence. Serum-free CM was used as a control. Growth of blood vessels on the CAM is shown (Fig. 2a). Expression of EGFP and EGFP-WWOX proteins and the endogenous WWOX and β-actin is shown (Fig. 2b–d). Expression of basic fibroblast growth factor (bFGF) protein is upregulated in BCC-expressing EGFP-WWOX (Fig. 2c, d). bFGF protein in secretion was almost doubled in BCC expressing EGFP-WWOX, compared to controls. By ELISA, significant upregulation of bFGF protein in cells expressing EGFP-WWOX is shown (Fig. 2e).

Human umbilical vein endothelial cells (HUVEC) were used to form capillary tubes on Matrigel. The conditional medium (CM) from EGFP-WWOX-expressing cells supported the capillary tube formation (Fig. 2f). Serum-free medium and the CM from EGFP-expressing cells had much less effect (Fig. 2f). As low as 2% serum in the medium, EGFP-WWOX-expressing cells secreted greater amounts of bFGF than control cells (Fig. 2g, h).

### Cells deficient in WWOX do not merge with parental cells and other WWOX-positive cells

**Fig. 3:**
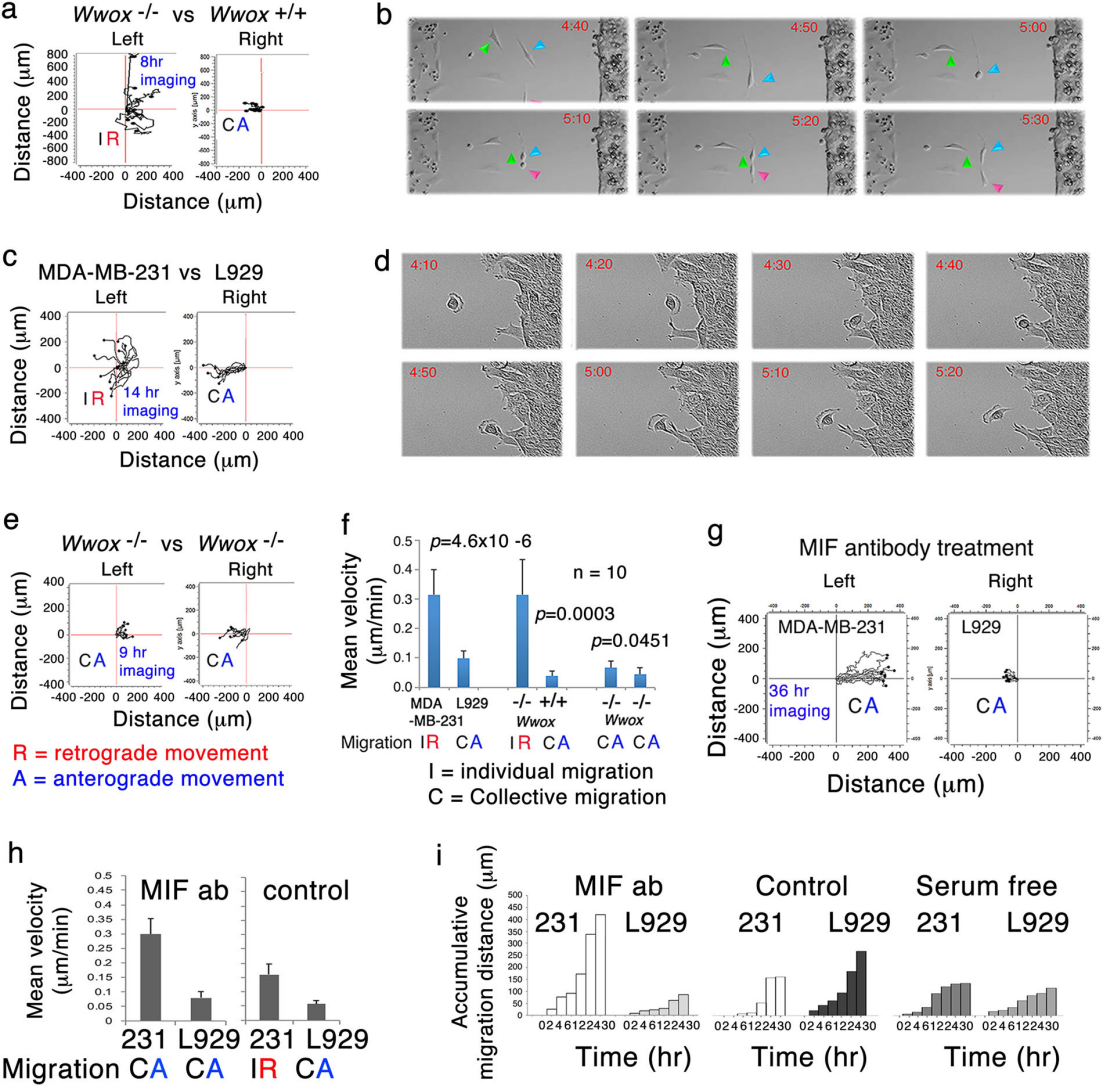
WWOX is a potent inhibitor of cell proliferation and migration. **a**, **b** MEF knockout *Wwox*^-/-^ cells (left) and wild type *Wwox*^+/+^ cells (right) were seeded, respectively, in each side of the culture-insert (*ibidi*) for 48 h. Upon removing the insert, cells from each side migrated to each other. When knockout cells moved closely to the wild type cells, they turned backward rapidly (i.e., retrograde migration). The migration patterns of 10 randomly selected cells are shown. Also, see Fig. S3 and Videos S1 and S2. **c**, **d** WWOX-negative human breast MDA-MB-231 cells (left) migrated in a retrograde manner upon encountering WWOX-positive murine L929 cells (right). Also, see Fig. S4 and Videos S3 and S4. **e** MEF knockout *Wwox*^-/-^ cells in both sides of the culture-insert underwent anterograde migration and grouped nicely together. Also, see Fig. S5 and Video S5. **f** Mean velocity=Averaged distance (μm) of the entire routes of 10 randomly selected cell traveled / time (min; *n* = 10, Student’s *t* test). **g**–**i** Anti-MIF antibody (1 μg/ml) was added to the coculture of MDA-MB-231 (or 231) and L929 cells for migration analysis by time-lapse microscopy. Mean velocity and accumulative migration distance were calculated. In controls, medium alone or serum-free condition was used

Methylation inhibitors 5-azacytidine and 5-aza-2′-deoxycytidine restore WWOX expression in promoter-hypermethylated cells, thereby inducing cell growth inhibition^32,33^. *Wwox* knockout MEF (mouse embryonic fibroblast) cells have loose cell-to-cell contacts^34^. Wild type MEF cells migrated collectively, while *Wwox* knockout MEF cells migrated individually. An equal amount of knockout *Wwox*^−/−^ and wild type *Wwox*^+/+^ MEF cells was seeded, respectively, in each side of the reservoirs of a culture-insert (from *ibidi*) with a distance of 500 μm (Fig. 3a, b, S3; Video S1, S2). 2% fetal bovine serum (FBS) was included in the coculture system to limit cell division. After 24 to 48 h in culture, culture-insert was removed and cells migrated to each other. *Wwox*-knockout cells migrated faster than the wild type cells, as determined by time-lapse microscopy^30,35^. When *Wwox*-knockout cells migrated to the wild type cells within 10–50 μM in distance, knockout cells extended their pseudopodia to probe the wild type and then quickly moved backward. Frequently, knockout cells underwent mitotic division post retrograde migration (Fig. 3b; Video S1, S2). One cell kept moving forward to the wild type MEF cells, and the other back to the home base. Knockout cells fail to merge with wild type, although knockout cells were derived from the wild type cells^34^.

When WWOX-negative human MDA-MB-231 cells encountered WWOX-positive murine L929 fibroblasts, MDA-MB-231 accelerated their individual anterograde migration toward L929, followed by retrograde movement (Fig. 3c, S4; Video S3). MDA-MB-231 may undergo mitotic division. L929 is sensitive to tumor necrosis factor (TNF)-mediated apoptosis^10^. Indeed, MDA-MB-231 cells may migrate toward L929 to have physical contacts and then left rapidly to return to their home base (Fig. 3d). Both types of cells could sense the presence of each other from a distance, suggesting that cell-derived cytokines affect the cell migratory behavior.

When MEF *Wwox*^−/−^ cells met each other, they moved slowly and steadily forward, and merged nicely (Fig. 3e, S5; Video S5). Similar results were observed by testing wild type MEF (Video S11), L929, or MDA-MB-231 cells alone (data not shown). Their status of migration, either individual or collective, is shown (Fig. 3f). Indeed, when WWOX-expressing L929 or wild type MEF cells encountered WWOX-negative MDA-MB-231 or *Wwox* knockout MEF cells, both cells had accelerated rates of migration (Fig. S6). That is, upon encountering WWOX-negative cells, WWOX-expressing cells increase their migration rates and distance.

### Inhibition of MIF/ERK signaling leads MDA-MB-231 to migrate collectively forward and allows their physical contacts with L929

To examine the involvement of cytokines, monoclonal antibody against macrophage migration inhibitory factor (MIF) was included in the coculture of MDA-MB-231 and L929. MDA-MB-231 migrated collectively without retrograde movement, and merged with L929 (Fig. 3g; Video S6). Compared to controls, mean velocity and accumulative migration distance for MDA-MB-231 were almost doubled (Fig. 3h, i). In controls, medium alone, non-immune IgG, or serum-free condition failed to alter the individual and retrograde migration of MDA-MB-231. MIF participates in inflammation, immunodulation, tumor growth and metastatic potential^36^.

MIF signaling activates the downstream MEK/ERK signaling^37^. Inhibition of MEK by U0126 allowed shift of individual to collective migration in MDA-MB-231 (Fig. S7; Video S7, S8). Alternatively, MDA-MB-231 cells were pre-exposed to U0126 for 40 min followed by cell migration assay (Fig. S7; Video S8). U0126-treated MDA-MB-231 cells increased their speed of migration by one-fold (Fig. S7). When L929 cells were pretreated with U0126, the treated L929 cells did not increase the migration speed of MDA-MB-231 cells. No retrograde migration and cell death were observed for all experimental conditions (Fig. S7), suggesting that MIF-activated ERK signaling is needed for retrograde migration of MDA-MB-231 upon facing L929.

### Membrane ephrins are needed for the retrograde migration of MDA-MB-231 upon facing L929

**Fig. 4:**
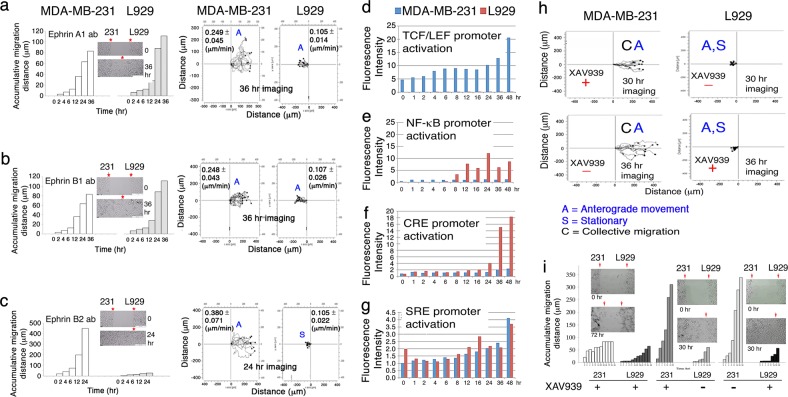
Eph/ephrin signaling and Wnt-dependent TCF/LEF promoter activation cause retrograde migration of MDA-MB-231 upon facing L929. **a**, **b** The coculture of MDA-MB-231 and L929 cells was added antibody (1 μg/mi) against ephrin A1 or B1, followed by time-lapse microscopy. Upon facing each other, MDA-MB-231 underwent collective and anterograde migration. Both the accumulative migration distance and the migrate velocity are shown. The starting points and the final cell merge point are marked (see red arrows). **c** In contrast, ephrin B2 antibody induced the anterograde migration of MDA-MB-231, along with increased migration velocity and distance. L929 migrated but remained largely in the same location. **d** MDA-MB-231 and L929 cells were transfected with an indicated promoter vector for 24 h. Time-lapse microscopy was then carried out for the cell migration toward each other. Rapid activation of Wnt/β-catenin-dependent TCF/LEF promoter occurred in MDA-MB-231 in less than 2 h, but not in L929. **e**, **f** When MDA-MB-231 moved approaching toward L929 in 12 to 48 h, activation of NF-κB and CRE promoters occurred in L929. **g** No apparent differences for the activation of SRE promoter in both cells. **h**, **i** When MDA-MB-231 or L929 were exposed to Wnt inhibitor XAV939 (10 μM) for 40 min, followed by preparing for time-lapse microscopy. MDA-MB-231 migrated collectively forward with an increased accumulative migration distance and an accelerated migration velocity. In contrast, L929 migration was significantly retarded. When MDA-MB-231 and L929 were treated with XAV939 (10 μM) for 40 min, followed by gently washing, replacing with fresh medium, and then starting time-lapse microscopy. Both cells migrated very slowly in an anterograde manner

The Eph/ephrin system participates in morphogenesis by governing cell position, collective migration and guidance, segregation, boundary formation, and cancer metastasis^38^. The cocultures of MDA-MB-231 and L929 were added with antibody against ephrin A1 or B1 for time-lapse microscopy. Both cells underwent anterograde and collective migration with enhanced velocities (Fig. 4a, b), compared to controls (Figs. 3 and 4i). In contrast, ephrin B2 antibody caused an accelerated anterograde migration of MDA-MB-231 cells, whereas L929 cells remained largely stationary (Fig. 4c). Ephrin B2 inhibits the signaling of ERK, JNK, p38, AKT and Stat3^39^. Together, signaling pathways via ephrins and MIF/ERK converge to ERK that allows the generation of the retrograde and individual migration of MDA-MB-231 upon facing L929.

### Activation of Wnt-dependent TCF/LEF promoter in MDA-MB-231 allows them to dodge L929

In the MDA-MB-231/L929 coculture, rapid activation of Wnt/β-catenin-dependent TCF/LEF promoter occurred within 4 h and reached a maximal extent in 48 h in MDA-MB-231, but not in L929 (Fig. 4d). Also, migrating MDA-MB-231 induced L929 to exhibit the NF-κB promoter activation in 12 h and last up to 48 h (Fig. 4e), and the CRE promoter activation between 24 and 48 h (Fig. 4f). Continuous activation of the SRE promoter in both cells started at 4 h and reached maximally at 48 h(Fig. 4g). AP1 promoter activation occurred in the beginning and lasted the entire course of the migration of knockout *Wwox* MEF toward wild type MEF cells (data not shown). In negative controls, MDA-MB-231 or L929 were seeded on both sides of the culture-insert and transfected with the aforementioned promoter vectors. No promoter activation was observed during cell migration (data not shown).

From the kinetics of promoter activation (Fig. 4d–g), we examined whether Wnt signaling contributes to retrograde migration of MDA-MB-231 upon facing L929. MDA-MB-231 or L929 cells were pretreated with Wnt inhibitor XAV939 (10 μM) for 40 min, followed by gentle washing and processing for time-lapse microscopy. MDA-MB-231 migrated collectively forward, and then merged with L929 (Fig. 4h, i). MDA-MB-231 accelerated their migration and traveling distance. In contrast, L929 cells remained largely stationary, and their migration suppressed (Fig. 4h, i). The event is similar to the observation using ephrin B2 antibody (Fig. 4c).

When MDA-MB-231 and L929 in coculture were exposed to XAV939 (10 μM) for 40 min, followed by time-lapse microscopy. Both cells slowly migrated in an anterograde manner, but without merge in 96 h (Fig. 4i). MDA-MB-231 migrated individually, whereas L929 moved collectively forward. Antibody against membrane APP (amyloid precursor protein) did not abolish the retrograde migration of MDA-MB-231 (data not shown). APP participates in the progression of Alzheimer’s disease and cancer^2^. Together, when Wnt signaling is turned off, MDA-MB-231 cells undergo collective and anterograde migration upon facing L929.

### TGF-β1 induces MDA-MB-231 to migrate forward in a collective manner upon facing WWOX-positive L929 or lung primary epithelial cells

We investigated whether TGF-β restricts retrograde migration. TGF-β family proteins control cell growth, extracellular matrix protein synthesis, and immune cell functions. TGF-β plays a dual role in cell growth and tumorigenesis. TGF-β inhibits mammary epithelial cell growth, and promotes epithelial-to-mesenchymal transition (EMT). Invasive cancer cells frequently overproduce TGF-β to promote growth and metastasis^40^.

**Fig. 5:**
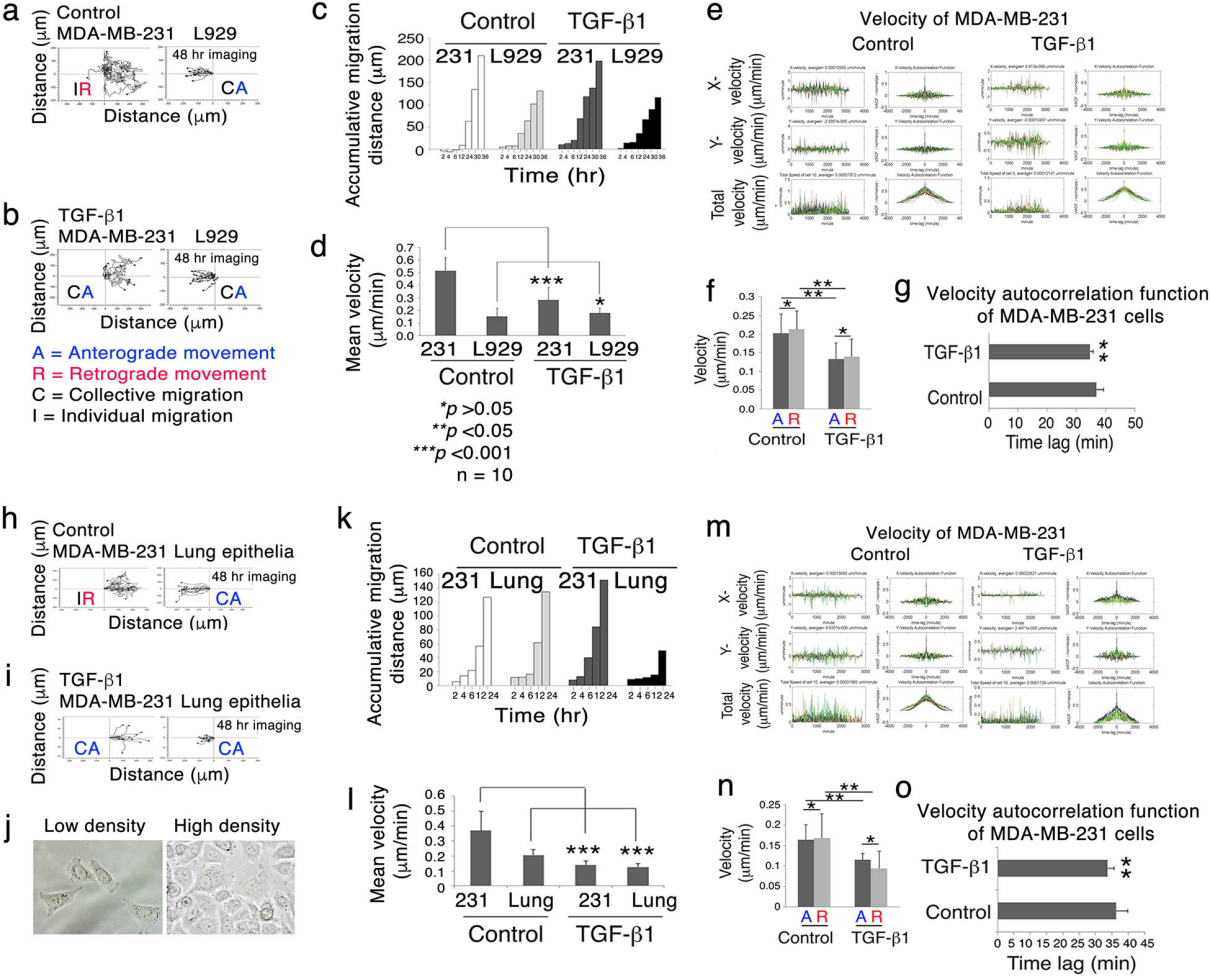
TGF-β1 restores the collective anterograde migration of MDA-MB-231 upon facing WWOX-positive L929 and lung primary epithelial cells. **a** MDA-MB-231 (left) and L929 cells (right) were seeded, respectively, in each side of a culture-insert (i*bidi*). After overnight incubation, time-lapse microscopy was carried out at 37°C with 5% CO2. MDA-MB-231 underwent retrograde migration upon encountering L929. **b** TGF-β1 (10 ng/ml) was included in the coculture and induced MDA-MB-231 to migrate collectively forward onto L929. **c**, **d** TGF-β1 suppressed the migration velocity of MDA-MB-231. The accumulative migration distance is shown. **e** Temporal correlation of the velocity was performed to randomly analyze the migratory behavior of 10 cells (left column). X, Y and total velocity were calculated. The velocities of cells in each time point are shown as peaks. Velocity Autocorrelation Function (VACF) is shown (right column). **f** Statistical analysis of migrating velocity, anterograde versus retrograde, of cells treated with or without TGF-β1. **g** TGF-β1 significantly suppressed VACF of MDA-MB-231. **h**–**j** TGF-β1 (10 ng/ml) induced MDA-MB-231 to undergo anterograde and collective migration upon facing WWOX-positive lung primary epithelial cells. **k**, **l** TGF-β1 suppressed the migration velocity of MDA-MB-231. The accumulative migration distance is shown. **m**–**o** In parallel, the analyzes and statistics of total velocity autocorrelation function are shown for MDA-MB-231 versus lung epithelial cells. For all experiments shown above, data are shown as mean ± standard deviation and n *=* 10 (Student’s *t* test)

TGF-β1, at 10 ng/ml, was added to the coculture of MDA-MB-231 and L929 cells. MDA-MB-231 migrated individually and moved backward upon facing L929 at a close range of <50 μm (Fig. 5a, b). TGF-β1 induced collective and anterograde migration of MDA-MB-231 and reduced their speed of migration (Fig. 5c, d).

To further validate the cell migration pattern, we utilized the Velocity Autocorrelation Function (VACF) analysis to determine the movement of MDA-MB-231 in facing L929 (Fig. 5e–g)^41^. In the random process, the correlation function reflects how smooth or wiggly a process is. The velocity of TGF-β1-treated MDA-MB-231 was lower than that of control (Fig. 5e, f). Total VACF was also significantly lower than control (Fig. 5e, g).

Metastatic cancer cells encounter WWOX-rich cells in the epithelia and endothelia of tissues and organs in vivo. We isolated lung epithelial cells from NOD-SCID mice. As expected, when MDA-MB-231 migrated toward the WWOX-positive lung epithelial cells, MDA-MB-231 moved individually forward and then migrated backward to their home base (Fig. 5h–l). TGF-β1 suppressed the retrograde migration and the velocity of MDA-MB-231 (Fig. 5i, l). The data were validated by VACF (Fig. 5l–n). Together, WWOX-negative metastatic cancer cells effectively avoid direct confronting with WWOX-positive cells in the microenvironment.

### WWOX-deficient cells induce redox activity and apoptosis of wild type cells

**Fig. 6:**
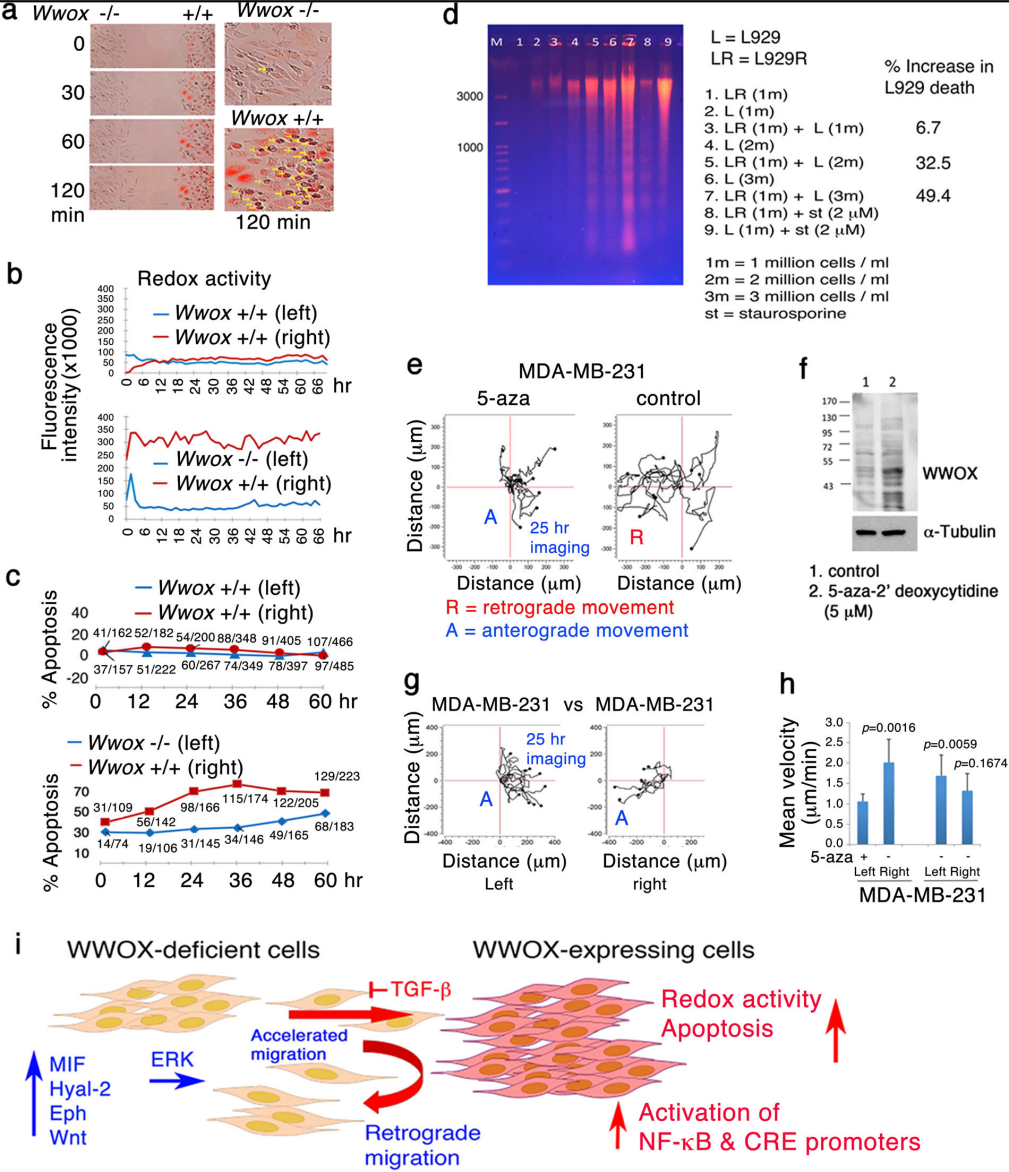
WWOX-negative cells rapidly induce redox activity and apoptosis of WWOX-positive cells. **a**–**c** By time-lapse microscopy, rapid upregulation of redox activity and apoptosis of wild type MEF cells were shown in the coculture of *Wwox* knockout and wild type MEF cells. **d** Direct mixing of WWOX-negative L929R cells with L929 for culturing 48 h at 37 °C led to apoptosis of L929 cells, as measured by DNA fragmentation analysis. Non-specific death of L929R cells was deducted from the total death of L929. **e**–**h** MDA-MB-231 cells were treated with methylation inhibitor 5-aza-2′ deoxycytidine (5-aza, 5 μM) for 5 days. 5-aza induced WWOX protein expression in MDA-MB-231, as determined by Western blot. The untreated parental cells underwent retrograde movement upon facing 5-aza-treated cells. In controls, both sides of untreated MDA-MB-231 migrated in an anterograde manner and merged with each other smoothly. Migration distance and velocity of cell migration are shown (*n* = 10, all samples versus 5-aza-treated cells; Student’s *t* test). Also, see Video S15. **i** A schematic model is shown for how WWOX-negtaive cells undergo retrograde migration upon facing WWOX-positive cells. WWOX-negative cells activate multiple pathways that may converge ERK, and this leads to evasion of WWOX-negative cells by retrograde migration and apoptosis of a portion of WWOX-positive cells. TGF-β abolishes the retrograde migration and apoptosis, and induces anterograde migration and merge of WWOX-positive and -negative cells

For survival WWOX-negative cells are able to kill the WWOX-positive cells. From a distance of 500 μm, *Wwox* knockout cells rapidly caused the upregulation of redox activity of wild type cells in <30 min, as determined using Redox Sensor Red CC-1 (Fig. 6a, b; Video S9, S10). Knockout cells exhibited little or no redox activity. Notably, wild type cells underwent apoptosis, as determined by uptake of propidium iodide in the nuclei (see yellow arrows; Fig. 6a, c; Video S9). In the wild type versus wild type controls, no increased redox activity and no apoptosis were observed (Fig. 6a–c; Video S11, S12). When TGF-β1 or antiserum against Hyal-2 was added to the coculture of the knockout and wild types cells, knockout cell-induced apoptosis of wild type cells was blocked (Fig. S9, S10). Similarly, human WWOX-negative breast MDA-MB-435S cells induced apoptosis of the wild type MEF cells, as both cells migrated closer (Video S13). Few MDA-MB-435S cells also underwent apoptosis (Video S13).

Similar observations were obtained when testing MDA-MB-231 versus L929 in cell migration, in the presence of 2% FBS in the medium. Many L929 underwent membrane blebbing and apoptotic death upon facing MDA-MB-231 from a distance (Fig. S10a; Video S3 and S4). Little or no death for MDA-MB-231 cells was observed. In the absence of serum, many more L929 cells died of apoptosis (Fig. S10b; Video S14). MIF antibody was added in the coculture of MDA-MB-231 and L929, and no cell death observed during migration (Fig. S10c; Video S14).

### TNF-resistant L929R cells induce apoptosis of TNF-sensitive L929 cells during coculture

We utilized TNF-resistant L929R cells for direct encounter with the parental L929 cells by coculturing in Petri dishes for 48 h. L929R cells are deficient in WWOX and were shown to migrate in a retrograde manner upon encountering L929 cells. By increasing the numbers of L929R cells, there was an increased extent of L929 cells undergoing apoptosis (Fig. 6d). The background death of L929R cells was deducted from the intensity in the L929/L929R coculture. Similar results were observed by time-lapse microscopy using L929 versus L929R. However, there is an increased extent of L929R apoptosis post 48 h in coculture, as measured by uptake and nuclear accumulation of propidium iodide (data not shown).

### Induction of WWOX in MDA-MB-231 cells by a methylation inhibitor allows the cells to fend off parental WWOX-negative cells

Hypermethylation of *WWOX* gene promoter blocks protein expression in human cancers^1–5^. We induced WWOX expression in MDA-MB-231 by treating with 5-aza-2′-deoxycytidine (5-aza) to suppress promoter methylation for 5 days. Increased WWOX expression was shown (Fig. 6f). These cells started to attach to each other and migrated in a collective manner with significantly reduced migration velocity, compared to untreated parental cells (Fig. 6e, h; Video S15). When parental MDA-MB-231 cells encountered the 5-aza-treated cells, the parental cells were unable to recognize and merge with the WWOX-restored cells (Fig. 6e, h; Video S15). The parental cells underwent retrograde migration. In controls, when untreated parental MDA-MB-231 cells met each other, they migrated in an anterograde manner and then became merged (Fig. 6g).

### The *N*-terminus of WWOX enables MDA-MB-231 cells to fend off parental WWOX-negative cells

Finally, MDA-MB-231 cells were stably transfected with an expression construct for EGFP-WW1/2 domains (including the *N*-terminal head and two adjacent WW domains). Both the MDA-MB-231-WW cells and the parental cells underwent anterograde migration and merged together (Fig. S10d). The data suggest that the *N*-terminus of WWOX is essential for controlling cell-to-cell recognition and merger.

## Discussion

We examined the following questions regarding cancer cell metastasis. First, how do metastatic cancer cells decide where and when to penetrate the capillary endothelium and adhere to a target organ? Second, does every metastatic cancer cell leave its solid-tumor home base voluntarily, or just get kicked out by parental cells? Third, why do the sequential DNA promoter activations occur during cancer cell encountering with the microenvironemnt? Finally, do metastatic cancer cells run away from the unfriendly microenvironment and meanwhile tackle organ target cells?

WWOX controls cancer cell migration and metastasis^5,16,17^. Loss of WWOX in triple-negative breast cancer cells leads to activation of the JAK2/STAT3 pathway for metastasis^16^. In the very early stage of cancer initiation, WWOX is upregulated and then reduced, so as to facilitate cancer growth^14,15^. Overexpressed WWOX exerts its proapoptotic pressure to cause skin BCC cells to secrete bFGF to support capillary microtubule formation, and this conceivably favors cancer cell growth and metastasis. Loss of WWOX, along with increased secretion of hyaluronan and hyaluronidases, frequently occurs in cancer cells at the late stage to facilitate metastasis. Thus, suppression of Hyal-2 by antibody limits cancer growth.

WWOX- cells struggle to survive in a microenvironment that is rich in WWOX+ cells. WWOX- cells can no longer recognize the parental cells or WWOX+ cells of the same or different species (using >15 pairs of cells tested thus far; unpublished). WWOX- cells fail to merge with WWOX+ cells. Upon close contact, WWOX- cells accelerate their individual backward migration to run away from WWOX+ cells (Fig. 6i). Also, they upregulate the redox activity of parental or WWOX+ cells for causing apoptosis. WWOX- cells utilize multiple signal pathways such as MIF, Hyal-2, Eph, and Wnt to converge to ERK, thereby undergoing retrograde migration. The observations were confirmed by specific blocking antibodies. MEK/ERK signaling is abolished by U0126. Membrane APP does not participate in the retrograde migration. While metastatic cancer cells secrete autocrine TGF-β to support their growth, exogenous TGF-β1 enables WWOX- and WWOX+ cells of the same or different species to merge, suggesting that autocrine TGF-β1 assists the WWOX- cells to compromise with WWOX+ cells.

Under the influence of murine L929, human MDA-MB-231 cells exhibit a rapid activation of the Wnt-mediated TCF/LEF promoter for accelerating their migration. Inactivation of the Wnt signaling leads MDA-MB-231 to successfully merge with L929. No MDA-MB-231-mediated L929 cell death is shown. WWOX inhibits the Wnt/β-catenin signaling pathway via binding with Dishevelled protein^31^.

While a portion of WWOX is anchored in the cell membrane by Hyal-2 and Ezrin^4,29,30^. Induction of WWOX expression by 5-aza in MDA-MB-231 allows them to undergo collective migration and repel parental MDA-MB-231 cells. Ectopic expression of the *N*-terminal head and the adjacent WW domains in MDA-MB-231 allows them to recognize WWOX-positive cells, suggesting that the indicated *N*-terminal region in WWOX participates in cell-to-cell recognition and connections.

When secreted MIF protein is blocked by antibody, the cell-to-cell recognition and merger among WWOX-positive and negative cells are restored. No cell death occurs, suggesting that MIF is involved in the initial sensing step that activates the machinery for the retrograde migration of WWOX- cells upon facing WWOX+ cells. Another scenario is that WWOX- cells rapidly induce the redox activity in the WWOX+ cells from a distance. This event may lead to apoptosis of WWOX+ cells. Apoptosis caused by overly increased redox activity and accumulated ROS has been well documented^42^. Upon facing WWOX-negative L929R, WWOX-positive L929 cells undergo apoptosis in 48 h, whereas there is a dramatic L929R apoptosis in another 48 h later. Similar results were observed with *Wwox* knockout MEF cells upon encountering with wild type MEF cells. The possibility that cytotoxic molecules released by WWOX-negative cells remain to be determined.

While WWOX-negative metastatic cancer cells lose recognition by their WWOX-positive parents, it is reasonable to assume that metastatic cancer cells get repelled from the parental cells. Metastatic cancer cells look for areas in a target organ, which has a low or deficient expression of WWOX. Successful attachment or docking to the target organ allows formation of a secondary tumor colony. WWOX- cancer cells release TGF-β to compromise with the WWOX+ cells.

Overall, WWOX- metastatic cancer cells face an unfriendly WWOX-rich microenvironment. WWOX- cells initiate multiple signaling pathways that may converge to ERK, so as to speed up the migration in a retrograde manner and evade WWOX+ cells. WWOX- cells secrete MIF for the initial sensing of WWOX+ cells from a distance. Subsequent activation of Hyal-2, Wnt and Eph/ephrin signaling pathways for converging to ERK is crucial for accelerated anterograde and then retrograde migration in WWOX- cells. Thus, inactivation of these pathways allows restoration of collective, anterograde migration of WWOX- cells and their merge with WWOX+ cells.

## Materials and methods

### Cell lines and cell culture

Cell lines, which were maintained in our laboratory and used in this study, included human breast MCF-7, MDA-MB-231 and MDA-MB-435s cancer cells, mouse 4T1 breast cancer cells, human skin basal cell carcinoma BCC cells, murine TNF-sensitive L929 fibroblasts, and TNF-resistant L929R cells (American Type Culture Collection)^10,14–17^. Mouse embryonic fibroblast (MEF) wild type cells and *Wwox* gene knockout cells were generated and maintained in RPMI-1640 medium supplemented with 10% fetal bovine serum^34^. All the cells were cultured at 37 °C in an incubator with 5% CO_2_/atmosphere.

### Chemicals, antibodies and Western blotting

Cell Tracker Red, Redox Sensor Red CC-1, and Phalloidin for F-actin staining were from Invitrogen. Antibodies against ephrin A1, B1, and B2, WWOX, and MIF were from Santa Cruz Biotechnology. APP antibody was from EMD Millipore. Methylation inhibitor 5-aza-2′ deoxycitidine and VAV939 Wnt inhibitor were from Sigma. Homemade antibodies against Hyal-2 and pY216-Hyal-2 were used, as described^21,29,30^.

### cDNA constructs and electroporation

The murine full-length *Wwox* cDNA was made in a mammalian expression pEGFP-C1 vector construct (Clontech)^11,16^. *Wwox* siRNA (*Wwox*si) was designed and cloned into pSuppressorNeo vector (Imgenex)^43^. Designed primers for WWOXsi#1 and #2 siRNAs, targeting human/murine *WWOX*/*Wwox* and human *WWOX*, respectively, were made^43^. Cells were electroporated twice with the indicated DNA constructs (200V, 50 ms) and cultured in medium containing 10% FBS overnight prior to carrying out experiments.

### Cell migration assay, promoter activation, and time-lapse microscopy

Cell migration assay was performed by using Culture-inserts (*ibidi*) for growing cells. A culture insert was placed onto a 35 mm dish, and equal numbers of cells (70 μl, 4 × 10^5^ cells) were seeded into the two reservoirs of the same insert with a 500 ± 50 μm gap. After overnight incubation at 37 °C/5% CO_2_, the insert was gently removed and the medium was changed to serum-free medium. The cell migration was imaged at an indicated time interval for 24 to 48 h using a NIKON TE2000-U microscope^20^. Also, cells were maintained in medium containing 2% FBS to minimize cell proliferation during migration. Cell migration was also analyzed either by counting the migrating cell numbers or by measuring the migrating cell areas. In addition, we used an inverted Olympus IX81 fluorescence microscope for carrying out time-lapse microscopy^20^. Cell migration rate was measured by cell migrating distance versus time. Single cell moving path was tracked using the NIH Image J manual tracking and chemotaxis and migration tool. Where indicated, promoter activation assay was carried out by time-lapse microscopy^20^. Assay kits for promoter functions driven by SMAD, NF-κB, TCF/LEF, SRE and CRE, respectively, were from SABiosciences. WWOX-negative MDA-MB-231 and WWOX-positive L929 cells (or other cell pairs) in each side of a culture-insert were transfected with a promoter construct using green fluorescent protein as a reporter by liposome-based Genefector (VennNova). Promoter activation was chased for 48 h by time-lapse fluorescence microscopy. Both positive and negative controls from the assay kit were also tested in each experiment.

### Velocity autocorrelation function (VACF)

VACF was calculated to confirm the treatment actually affected the cell migration for each initial condition^41^. The formula of calculation was:

The velocity is expressed as $\vec v\left( t \right) = \frac{{\left[ {\vec R\left( {t + \delta } \right) - \vec R\left( t \right)} \right]}}{\delta }$

The velocity autocorrelation function is defined as R(τ) = $\left\langle {\vec v\left( {t + \tau } \right) \cdot .\vec v\left( t \right)} \right\rangle$

$\vec R\left( t \right)$ means the position of a single cell tracking center. τ means the time interval of the normal diffusion. δ means the time interval of each frame. The maximum correlation value is 1. Lower the value means more uncorrelated. We used the temporal correlation of the velocity to randomly analyze the behavior of each cell migration using 10 cells. VACF is calculated by the aforementioned formula^41^.

### Tumorigenicity assay in nude mice

Nude mice were purchased from the National Laboratory Animal Center (Taipei, Taiwan), housed in a dedicated nude mouse facility with microisolator caging, and handled under a unidirectional laminar airflow hood. Control-transfected BCC cells (BCC/scramble), WWOX-transfected BCC cells (BCC/WWOX), WWOXsi-transfected BCC cells (BCC/WWOX1si) were trypsinized, washed with PBS, resuspended in PBS, and adjusted to a concentration of 5 × 10^5^ cells/100 μl in PBS. Cell transfectants were implanted subcutaneously on the dorsal flank of 5-week-old mice (5 animals per clone). Tumor volume was measured (3 per group) twice per week^21,22^. 8 weeks later mice were sacrificed, and tumors removed and weighed. Tukey’s multiple comparison test was used to compare the differences in tumor growth rate and tumor weight. A segment of tissue was excised and fixed in 10% neutral buffered formalin. Where indicated, an aliquot of Hyal-2 or control antiserum was injected via tail veins once per week for 3 weeks, followed by inoculating with an indicated cancer cell line.

### Reverse transcription-polymerase chain reaction (RT-PCR)

To quantify the relative *WWOX* mRNA levels in BCC cells, RT-PCR was carried out to amplify the exon 9 of *WWOX* mRNA transcripts using specific primers as follows: WWOX forward, 5′-AAAACGACTATTGGGCGATG-3′ WWOX reverse, 5′-GTGTTGGAGGGACATTTG GA-3′ ß-actin forward, 5′-AGCGGGAAATCGTGCGTG-3′ ß-actin reverse, 5′-CAGGGTACATGGTGGTG-3′. The presence of exon 8–9 in the mRNA indicates presence of a full-length transcript. The PCR products were separated in 1.5% agarose gels, analyzed with a UV transilluminator, and scanned with a densitometer (ONE-Dscan 1.33 software, Scanalytics, Fairfax, VA). The ratios of relative mRNA densities among BCC/scramble, BCC/WWOX and BCC/WWOXsi groups were calculated.

### Preparation of CM

BCC/WWOX, BCC/WWOXsi, or BCC/WWOX scramble cells were plated in 1 ml culture medium without serum at 2 × 10^5^ cells per well in 24-well 18 mm culture dishes. The culture supernatants were collected 24 h later and centrifuged sequentially at 12,500 × *g* with Microcon YM-3 centrifugal filter devices (cutoff molecules smaller than 3000Da; Millipore) for 10 min to obtain a 10-fold concentrate culture supernatant.

### CAM assay

Nine-day-old fertilized White Leghorn chicken eggs were incubated at 37 °C at constant humidity. On incubation at day 3, a square window was opened on the shell and sealed with a glass. On day 11, 1 mm^3^ filter papers loaded with 30 μL CM were implanted on top of the CAM. Capillary tube formations were examined 3 days later, when the angiogenic response peaked. The blood vessels entering the paper were recognized macroscopically and photographed.

### In vitro capillary tube formation on Matrigel

Human umbilical veins were collected with informed consents. The HUVEC capillary tube formation was evaluated as follows:. In total, 24-well 18 mm tissue culture dishes were coated with Matrigel basement membrane matrix (300 μl/well) (Becton-Dickinson) at 4 °C and allowed to polymerize at 37 °C for at least 30 min. The HUVECs (5 × 10^4^ cells/well, in 24-well 18 mm tissue culture dishes) were grown in a final volume of 0.4 ml culture medium containing 150 μl M199 (GibcoBRL) and 250 μl CM. After 6-h incubation, tube formation was observed through an inverted, phase-contrast photomicroscope, then photographed and counted. The number of tube formations was measured by counting the number of tube-like structures formed by connected endothelial cells in five randomly selected 9.7 mm^2^ microscopic fields. The assay was performed in triplicate.

### Enzyme immunoassay (ELISA)

The bFGF levels of the cell culture supernatants were determined by using commercially available ELISA kits (R&D Systems) according to the manufacturer’s instructions. Each measurement was repeated in triplicates, and the average value was recorded as picogram per ml.

### Statistical analysis

Data were analyzed by Student’s *t* test among controls and tested groups using Microsoft excel. Data were expressed as mean±S.D., where *p* < 0.05 was considered significant.

## Supplementary information

Supplemental Materials

Video S1. Migration of MEF wild type Wwox+/+ and knockout Wwox-/- cells in co-culture determined by time-lapse microscopy (low density)

Video S2. Migration of MEF wild type Wwox+/+ and knockout Wwox-/- cells in co-culture determined by time-lapse microscopy (high density)

Video S3. Migration of WWOX-negative MDA-MB-231 and WWOX-positive L929 cells in co-culture by time-lapse microscopy

Video S4. Migration of WWOX-negative MDA-MB-231 and WWOX-positive L929 cells in co-culture by time-lapse microscopy - touch down and kickout

Video S5. Migration of MEF Wwox-/- cells by time-lapse microscopy

Video S6. Inhibition of MIF by antibody abolishes the retrograde migration of MDA-MB-231 upon facing L929

Video S7. Inhibition of MEK/ERK by U0126 abolishes the retrograde migration of MDA-MB-231 upon facing L929

Video S8. Inhibition of MEK/ERK by U0126 abolishes the retrograde migration of MDA-MB-231 upon facing L929 (treatment of MDA-MB-231 only)

Video S9. Knockout Wwox-/- MEF cells dramatically upregulate the redox activity in wild type MEF cells from a remote distance (merged channels)

Video S10. Knockout Wwox-/- MEF cells dramatically upregulate the redox activity in wild type MEF cells from a remote distance (red channel)

Video S11. Wild type versus wild type MEF cells (merged channels): Redox activity in red

Video S12. Wild type versus wild type MEF cells (red channel): Redox activity in red

Video S13. MDA-MB-435s versus wild type MEF cells

Video S14. MDA-MB-231 cells induce a greater extent of L929 apoptosis under serum-free conditions

Video S15. Restoration of WWOX in MDA-MB-231 allows them to fend off WWOX-negative parental cells

Video S16. Ectopic expression of the N-terminus of WWOX allows MDA-MB-231 to merge with L929

Supplemental Video Legends
