# Supplementary material for: Strategies by which WWOX-deficient metastatic cancer cells utilize to survive via dodging, compromising, and causing damage to WWOX-positive normal microenvironment
Source: Cell Death Discov. 2019 May 21;5:97. doi: 10.1038/s41420-019-0176-4 (PMC6529460; doi:10.1038/s41420-019-0176-4)
Supplement: Supplementary file 1 — Supplemental Materials [file 41420_2019_176_MOESM1_ESM.pdf]

Supplemental Information

Fig. S1. **Hyal-2 antibody blocks cancer growth in mice.**
a-c NOD-SCID or nude mice received via tail veins with an aliquot of Hyal-2 or pY216-Hyal-2 antiserum (10µl in 90µl PBS) (**a,c)** or 2 µg Hyal-2 IgG or normal rabbit serum IgG (in 100µl PBS) (**b**), in 3 consecutive weeks or days. A week later, mice were subcutaneously injected with B16F10 or BCC cells (2 million cells in each flank). A representative data from two experiments is shown. The data support the observations in Fig. 1.

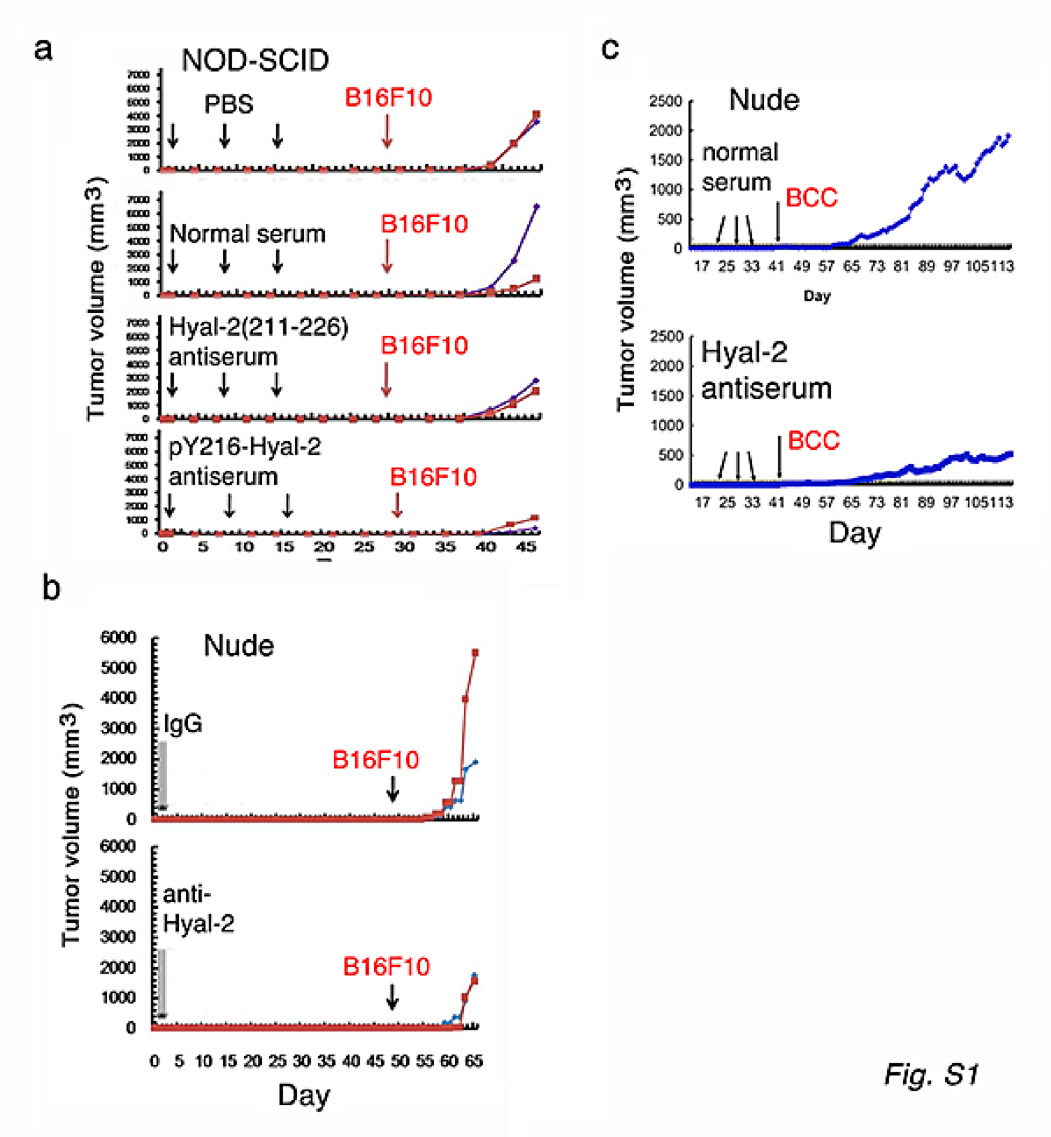

Fig. S1

Fig. S2. **Hyal-2 antibody increases cancer survival in mice.**
Ten BALB/c mice were inoculated subcutaneously with syngeneic breast cancer 4T1 cells in both sites of the flanks. When the tumors grew up to approximately 150 cubic mm 10 days later, each control mouse (5 in total) received via tail vein injections with 100 μl diluted normal rabbit serum (40 μl diluted in 60 μl PBS) for 3 indicated times. In the experimental group (5 in total), each mouse received via tail vein injections with 100 μl diluted rabbit antiserum against pY216-Hyal-2 (40 μl diluted in 60 μl PBS). Mouse survival is shown in a Kaplan-Meier survival curve. This data support the observations in Fig. 1.

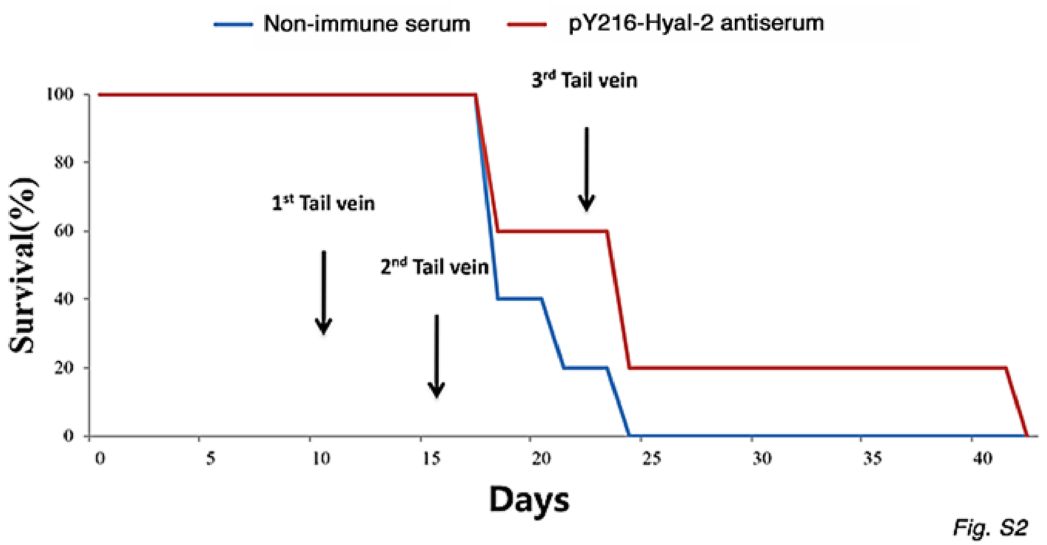

Fig. S2

Fig. S3. Time-lapse microscopy of MEF cell migration.

MEF knockout *Wwox*$^{-/-}$ (left) and wild type *Wwox*$^{+/+}$ cells (right) seeded, respectively, in each side of a culture-insert (*ibidi*). After overnight incubation, the insert was gently removed, and the medium replaced with fresh 2% FBS-containing medium. Time-lapse microscopy was carried out at 37°C with 5% CO_2. Each picture frame was taken per 10 min. When knockout cells migrated forward to a very close contact with the wild type cells, they moved backward (see blue arrows) and then divided in most cases. Data is derived from Video S1 and is linked to Fig. 3a.

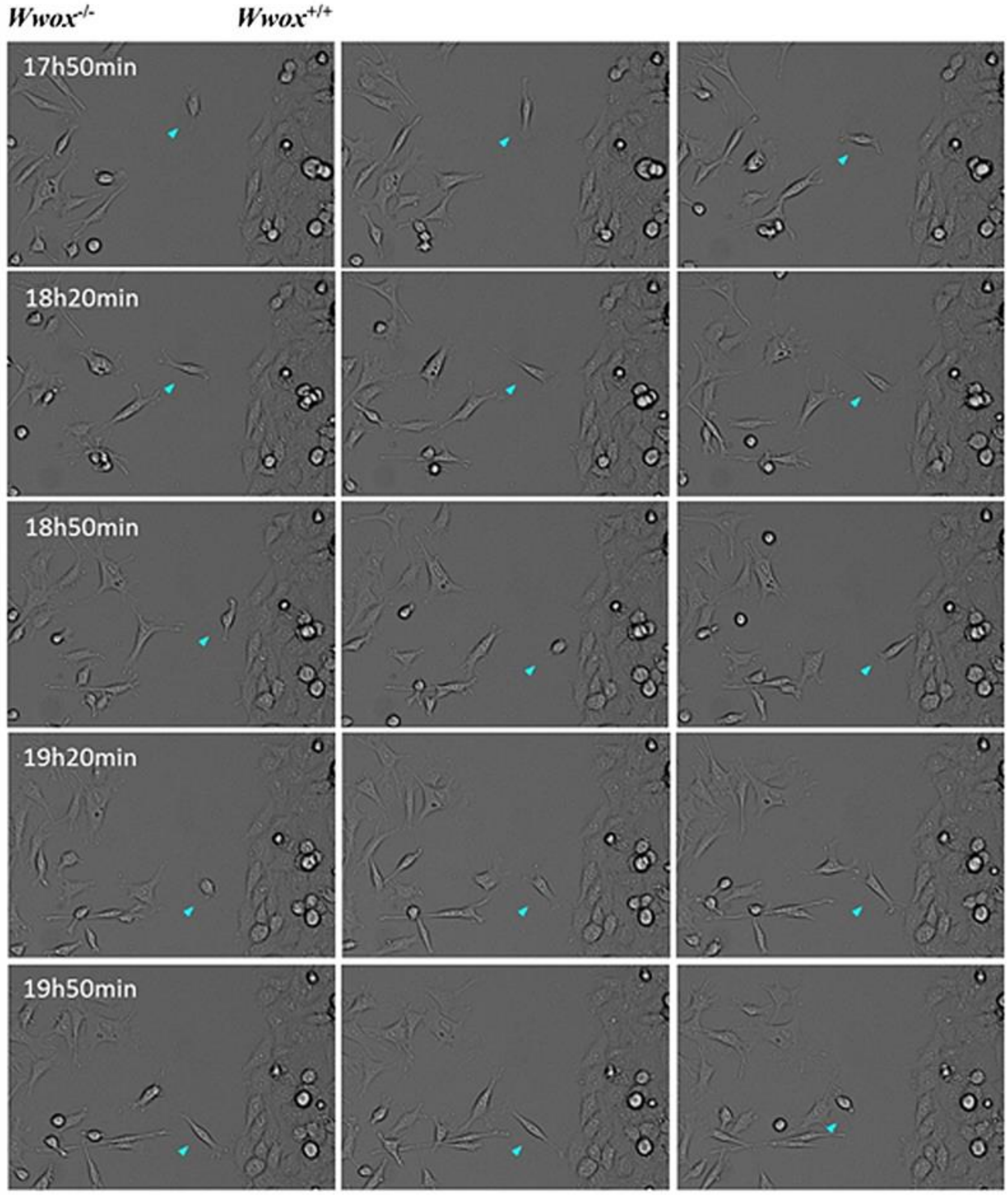

Fig. S3

Fig. S4. Time-lapse microscopy of *WWOX*-deficient MDA-MB-231 cells versus *Wwox*-expressing L929 cells in migration assay.
MDA-MB-231 (left) and L929 (right) cells were seeded, respectively, in each side of a culture-insert (*ibidi*). MDA-MB-231 cells moved forward and then backward upon encounering L929 cells (red arrows). Quite frequently, during encountering with L929, the migrating MDA-MB-231 cell divided into two cells (yellow arrows). Each picture frame was taken per 10 minutes. The data is derived from Video S3 and is linked to Fig. 3c.

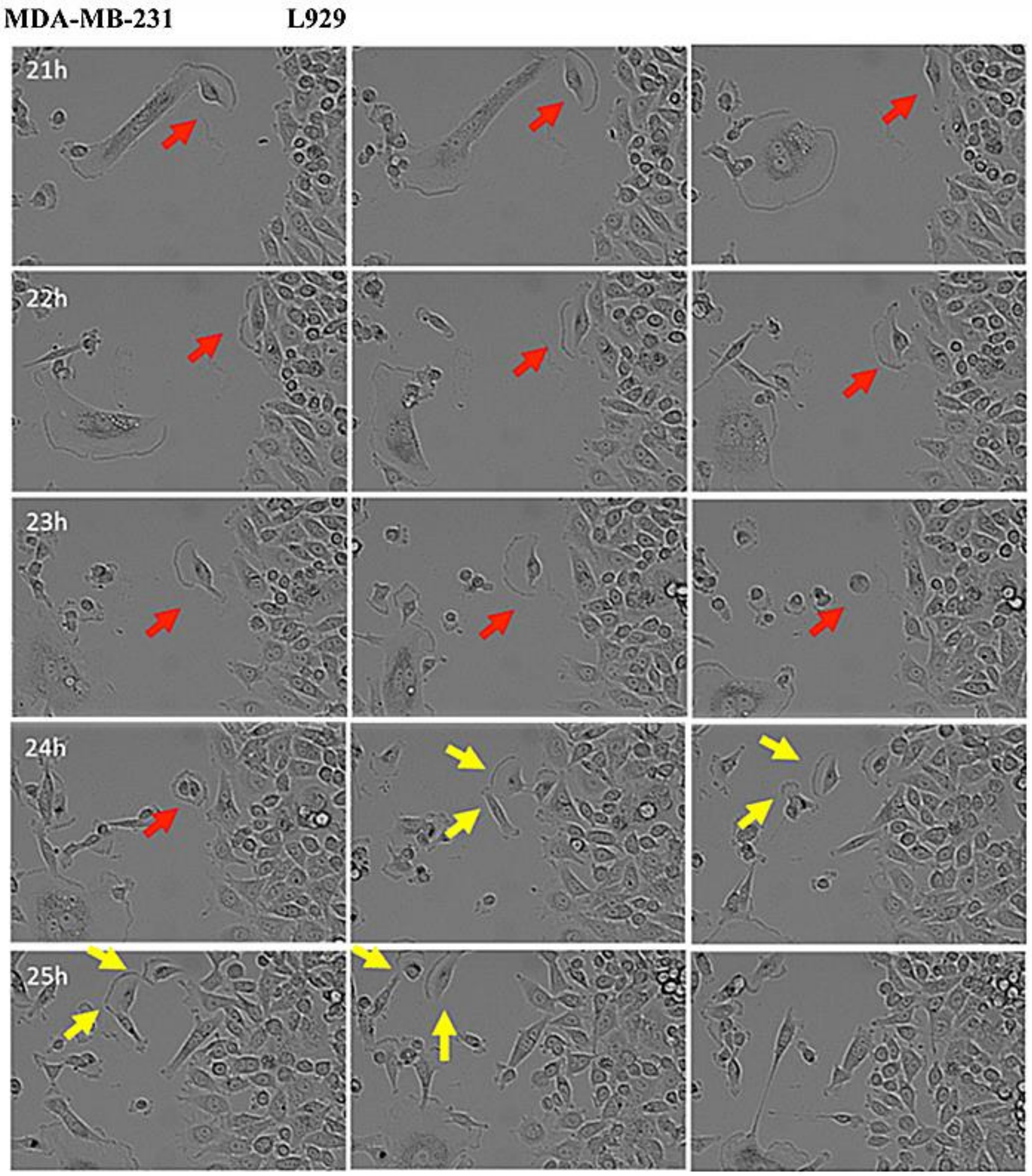

Fig. S4

Fig. S5. Time-lapse imaging of *Wwox*-knockout MEF migration.
When MEF *Wwox*$^{-/-}$ cells (left and right) met, they adhered to each other and grouped together firmly (yellow arrows). Each picture frame was taken per 10 minutes. The data is derived from Video S5 and is linked to Fig. 3e.

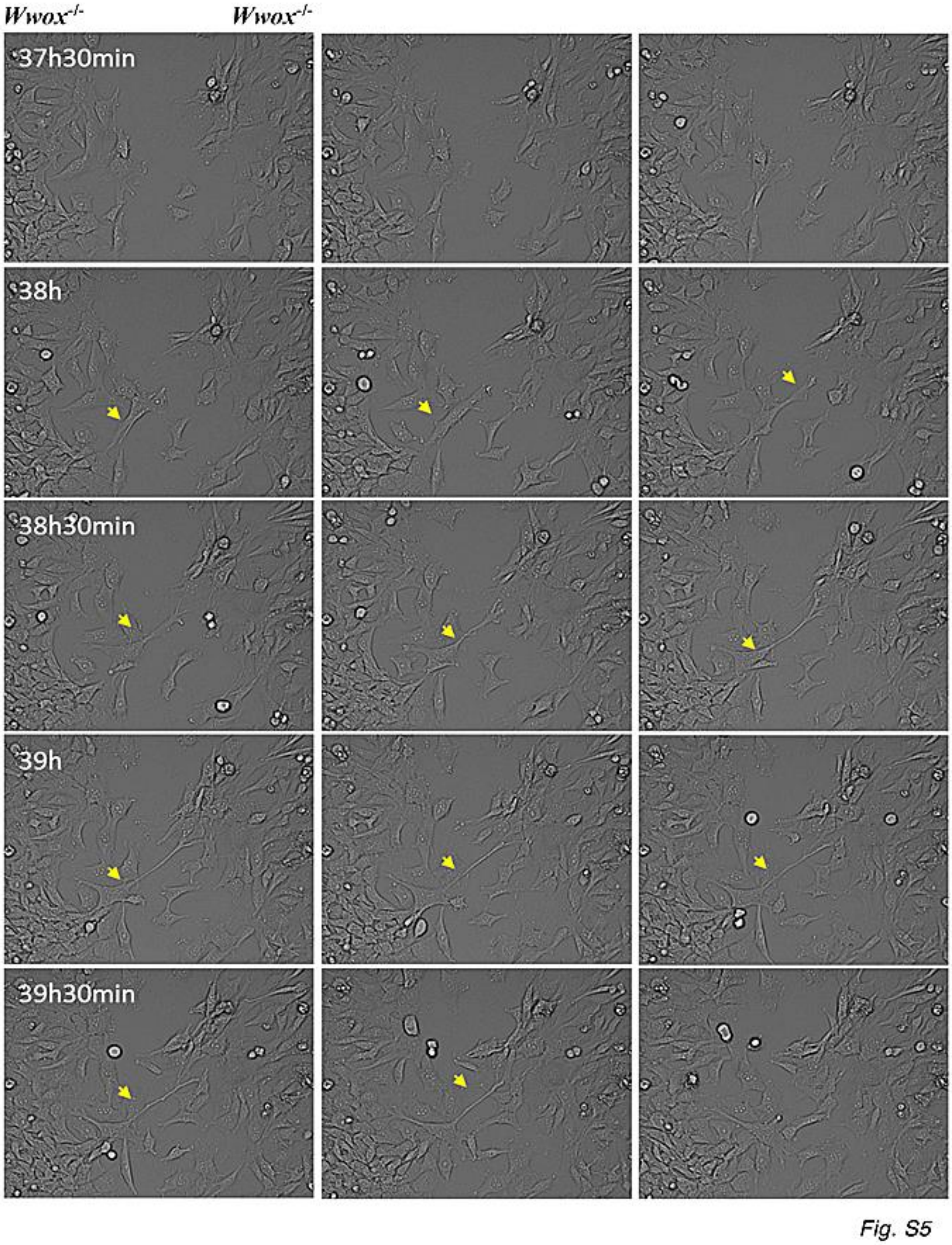

Fig. S6. Enhanced migration occurs when *Wwox*-positive cells encounter *Wwox*-negative cells.

Time-lapse migration assay was performed. Cell migration was analyzed by measuring the migrating distance versus time (n = 10). When two distinct cell types, expressing with or without WWOX, encountered each other, both cells had accelerated migration velocities and accumulated distance.

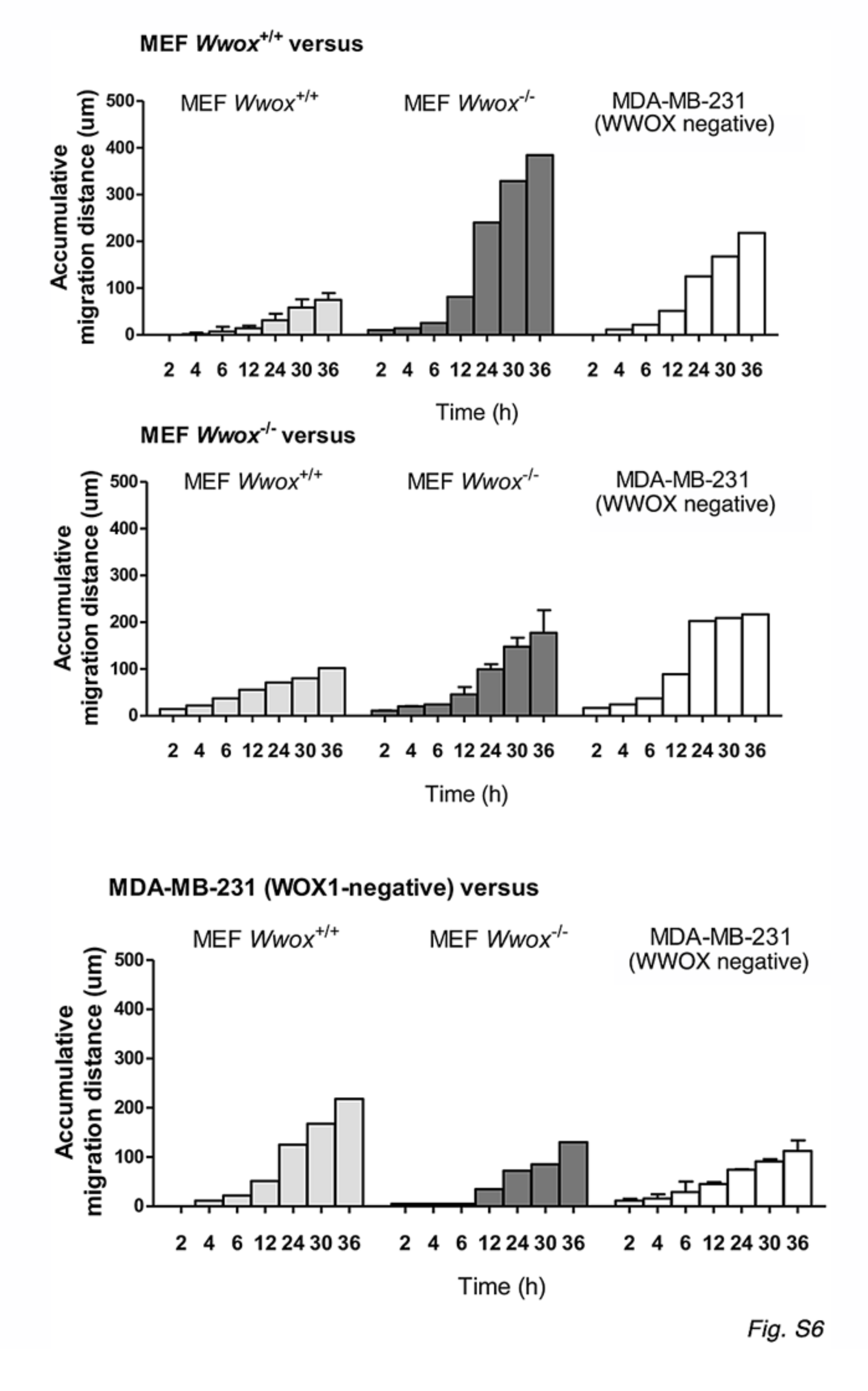

Fig. S7. Inhibition of MEK/ERK signaling allows MDA-MB-231 cells to migrate collectively forward and physically merge with L929.

a Time-lapse microscopy was carried out for the coculture of MDA-MB-231 and L929 cells. MEK inhibitor U0126 (10 μM) was added to the coculture during migration for time-lapse microscopy. Alternatively, MDA-MB-231 or L929 cells were treated with U0126 for 40 min, followed by washing and adding RPMI medium supplemented with 2% FBS for the migration assay. All MDA-MB-231 and L929 migrated collectively in anterograde manners. C= collective migration. **b** Representative micrographs show the collective migration of MDA-MB-231 versus L929 cells. U0126 was included during the cell migration. Also, see Video S7. **c** Representative micrographs show the collective migration of U0126-pretreated MDA-MB-231 versus L929 cells. Also, see Video S8.

a

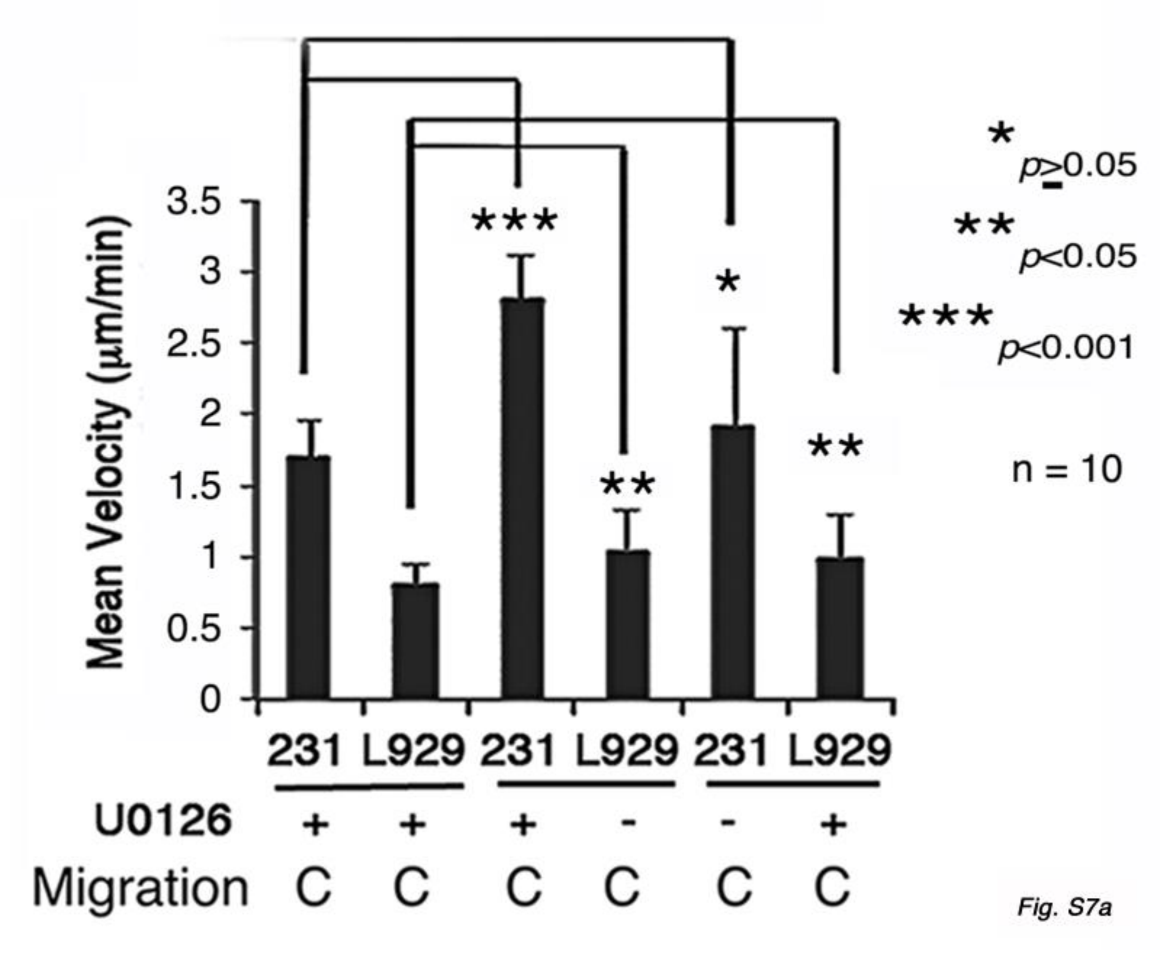

b

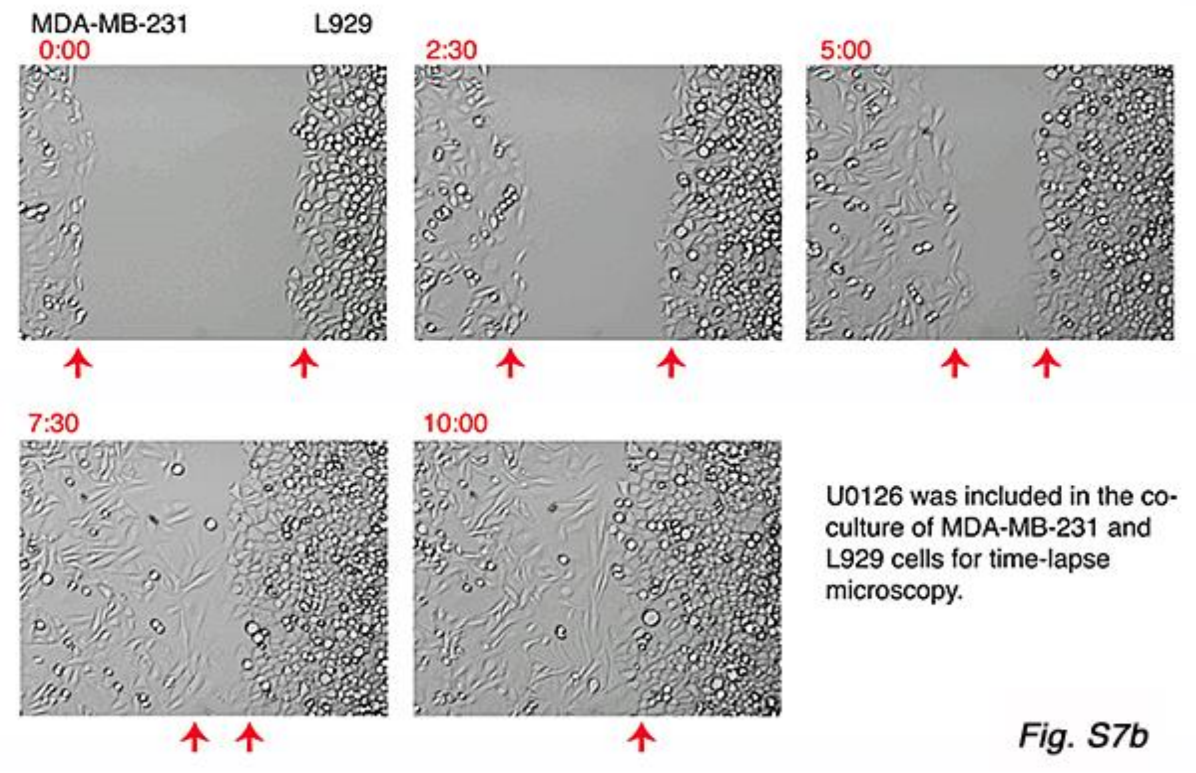
MDA-MB-231
L929
0:00
2:30
5:00
7:30
10:00
U0126 was included in the co-culture of MDA-MB-231 and L929 cells for time-lapse microscopy.
Fig. S7b

c

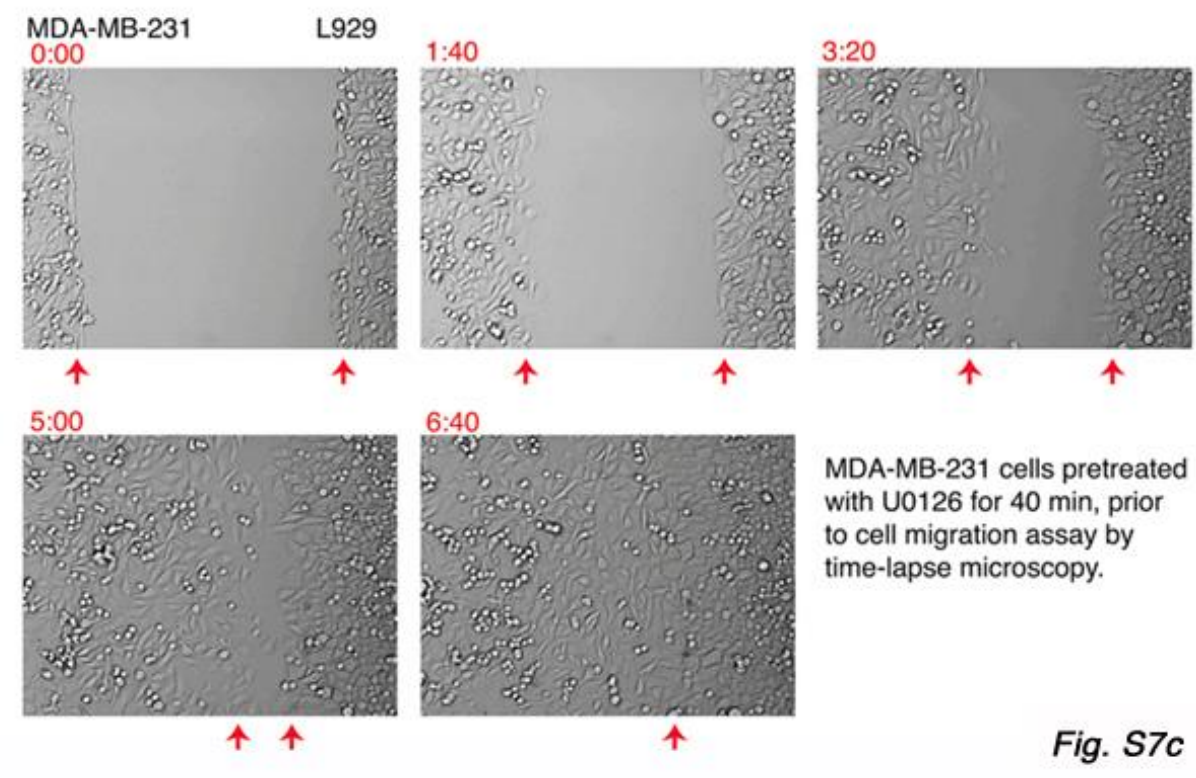
MDA-MB-231
L929
0:00
1:40
3:20
5:00
6:40
MDA-MB-231 cells pretreated with U0126 for 40 min, prior to cell migration assay by time-lapse microscopy.
Fig. S7c

Fig. S8. Hyal-2 antibody blocks cell migration-induced apoptosis.

Hyal-2 antiserum (4 μl/ml) was added to the coculture of *Wwox* knockout and wild type MEF cells for measuring migration by time-lapse microscopy. No apparent cell death was observed. Similar results were observed when *Wwox* knockout versus *Wwox* knockout cells in the presence of Hyal-2 antiserum (data not shown).

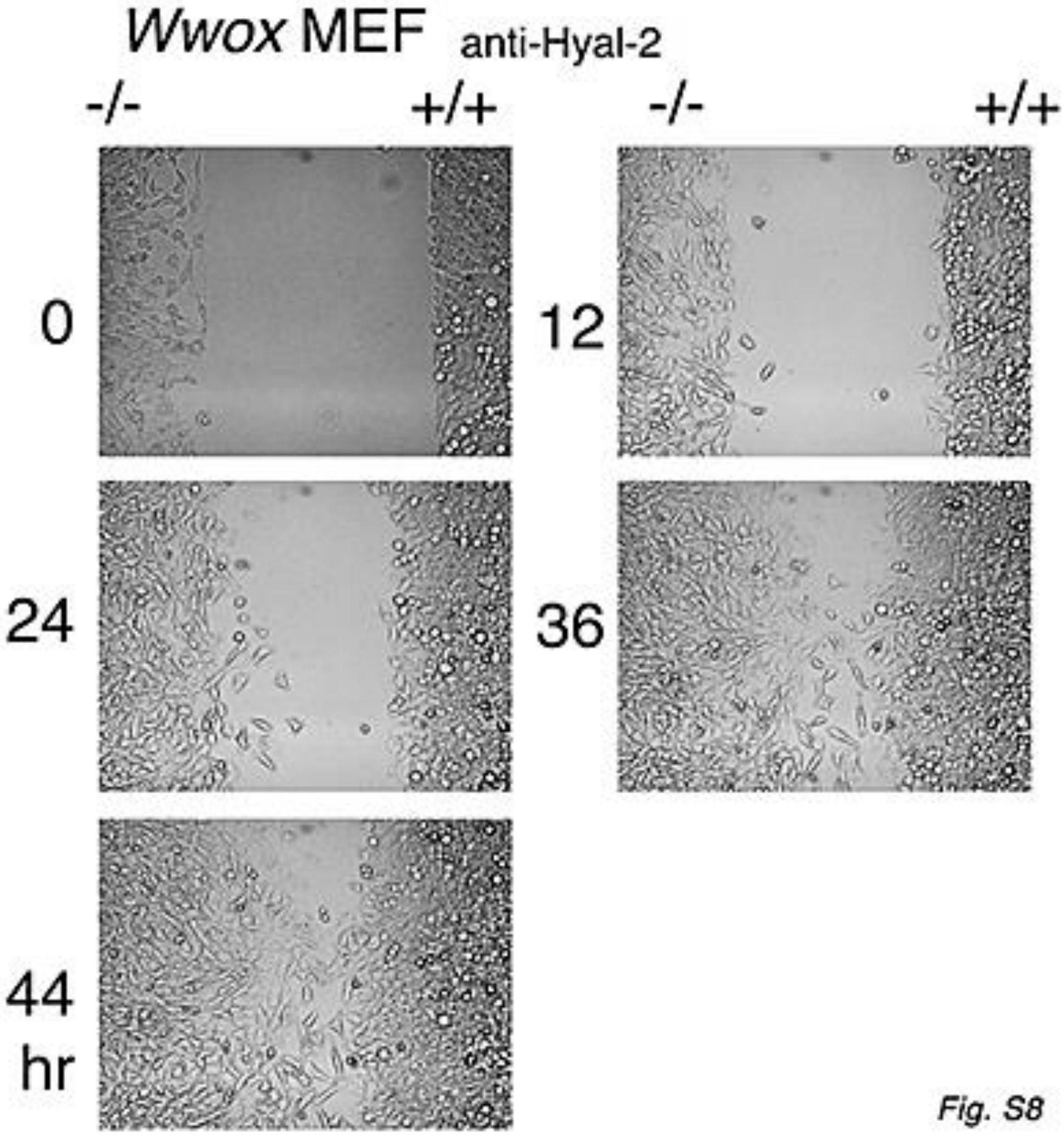

Fig. S9. TGF-β1 blocks cell migration-induced apoptosis.

TGF-β1 (1 ng/ml) was added to the coculture of *Wwox* knockout and wild type MEF cells for measuring migration by time-lapse microscopy. No apparent cell death was observed. Similar results were observed when *Wwox* knockout versus *Wwox* knockout cells in the presence of TGF-β1 (data not shown).

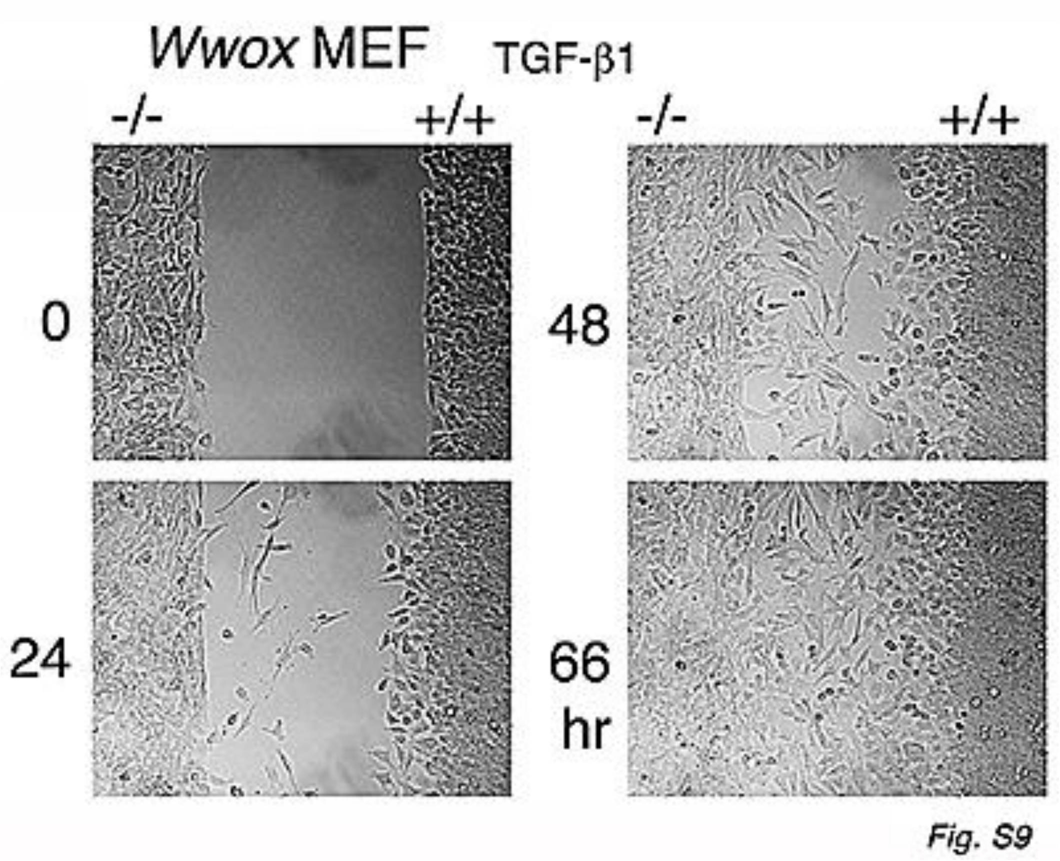

Fig. S10. Cell migration-induced apoptosis.
a Under normal cell migration coculture system using 2% FBS, MDA-MB-231 induced apoptosis of few L929 cells (red arrows). Also, see videos S3 and S4. **b** Under serum-free conditions, MDA-MB-231 induced greater numbers of L929 cells to undergo apoptosis (see Video S14). **c** MIF antibody (1 mg/ml) was included in the coculture of MDA-MB-231 and L929 cells. No cell death was observed during migration. **d** MDA-MB-231 were transfected with an expression construct for EGFP-WW1/2 domains (the *N*-terminal head and the adjacent two WW domains). Both MDA-MB-231-WW and L929 cells underwent anterograde migration and finally became merged. All cells were stained with Cell Tracker Red. Also, see Video S16. Red arrows show the cell migration fronts and their merge at hour 24.

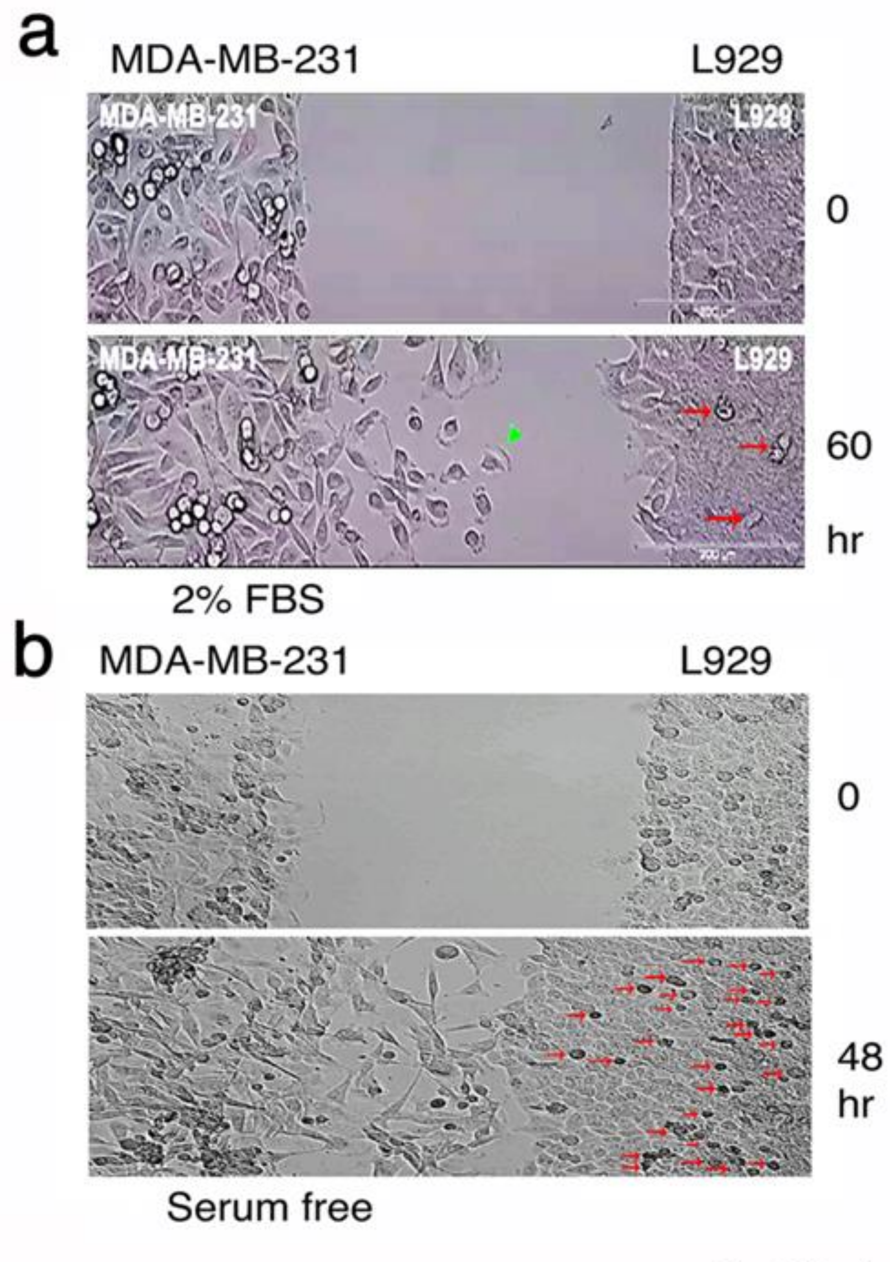

Fig. S10a,b

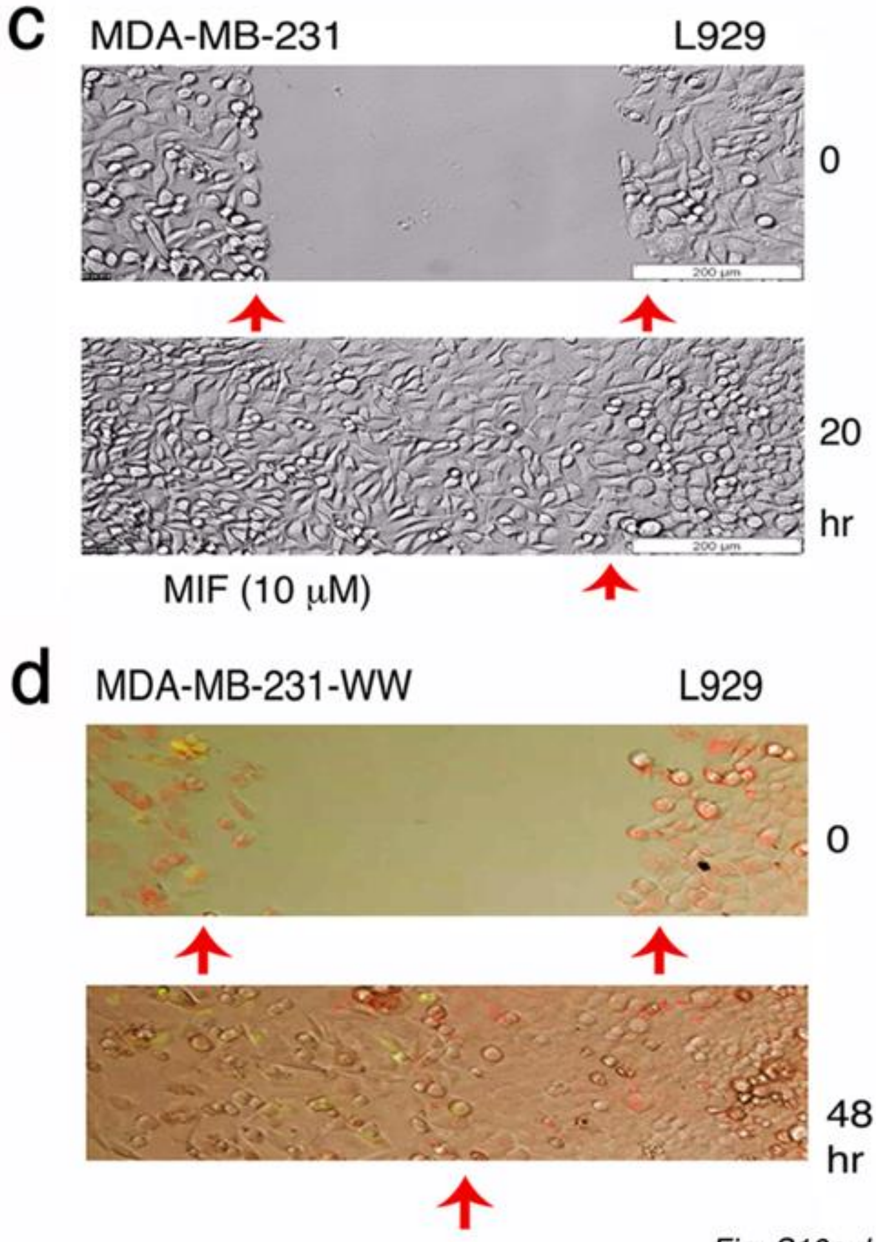
c
MDA-MB-231
L929
0
200 μm
20
hr
200 μm
MIF (10 μM)
d
MDA-MB-231-WW
L929
0
48
hr

Fig. S10c,d
