# Supplementary material for: Strategies by which WWOX-deficient metastatic cancer cells utilize to survive via dodging, compromising, and causing damage to WWOX-positive normal microenvironment
Source: Cell Death Discov. 2019 May 21;5:97. doi: 10.1038/s41420-019-0176-4 (PMC6529460; doi:10.1038/s41420-019-0176-4)
Supplement: Supplementary file 18 — Supplemental Video Legends [file 41420_2019_176_MOESM18_ESM.pdf]

Supplemental Video Legends

Video S1. Migration of MEF wild type *Wwox*$^{+/+}$ and knockout *Wwox*$^{-/-}$ cells in co-culture determined by time-lapse microscopy (low cell density).

MEF *Wwox*$^{-/-}$ cells (left) and *Wwox*$^{+/+}$ cells (right) were co-cultured for 24 and 48 hr in culture-inserts (*ibidi*), prior to performing time-lapse microscopy at 37°C with 5% CO_2. Low cell density is shown in S3. Each picture frame was taken per 10 minutes. These original video data are linked to Figure 3a and b.

Video S2. Migration of MEF wild type *Wwox*$^{+/+}$ and knockout *Wwox*$^{-/-}$ cells in co-culture determined by time-lapse microscopy (high cell density)..

MEF *Wwox*$^{-/-}$ cells (left) and *Wwox*$^{+/+}$ cells (right) were co-cultured for 24 and 48 hr in culture-inserts (*ibidi*), prior to performing time-lapse microscopy at 37°C with 5% CO_2. The cell density is higher than the experiment shown in Video S1. Each picture frame was taken per 10 minutes. These original video data are linked to Figure 3a and b.

Videos S3. Migration of WWOX-negative MDA-MB-231 and WWOX-positive L929 cells in co-culture by time-lapse microscopy (low cell density).

MDA-MB-231 cells (left) and WWOX-positive L929 cells (right) were co-cultured overnight in a culture-insert (*ibidi*), followed by performing time-lapse microscopy at 37°C with 5% CO_2. Each picture frame was taken per 10 minutes. These original video data are linked to Figure 3c, d, and S4 .

Videos S4. Migration of WWOX-negative MDA-MB-231 and WWOX-positive L929 cells in co-culture by time-lapse microscopy – single cell touch down and release

MDA-MB-231 cells (left) and WWOX-positive L929 cells (right) were co-cultured overnight in a culture-insert (*ibidi*), followed by performing time-lapse microscopy at 37°C with 5% CO_2. Note that a single MDA-MB-231 cell touched down the L929 cells, and then either got kicked out or escaped quickly. Each picture frame was taken per 10 minutes. These original video data are linked to Figure 3c, d, and S4 .

Video S5. Migration of MEF *Wwox*$^{-/-}$ cells by time-lapse microscopy.

MEF *Wwox*$^{-/-}$ cells were seeded in both chambers of a culture-insert (*ibidi*) and cultured overnight, prior to performing time-lapse microscopy at 37°C with 5% CO_2. Each picture frame was taken per 10 minutes. These original video data are linked to Figure 3c and S5.

Video S6. Inhibition of MIF by antibody abolishes the retrograde migration of MDA-MB-231 upon facing L929.

Antibody against MIF (1 μg/ml) was added to the coculture of MDA-MB-231 cells (left) and WWOX-positive L929 cells (right). MDA-MB-231 migrated forward collectively and merged with L929. Each picture frame was taken per 10 minutes. This data is linked to Figure 3g-i.

Video S7. Inhibition of MEK/ERK by U0126 abolishes the retrograde migration of MDA-MB-231 upon facing L929.

MEK inhibitor U0126 (10 μM) was added to the coculture of MDA-MB-231 cells (left) and WWOX-positive L929 cells (right) for 4 hr, followed by washing and preparing for time-lapse microscopy. MDA-MB-231 migrated forward in a collective manner, and merged with L929. Each picture frame was taken per 10 minutes. No cell death occurred for both sides. This data is linked to Figure S7a and b.

Video S8. Inhibition of MEK/ERK by U0126 abolishes the retrograde migration of MDA-MB-231 upon facing L929 (treatment of MDA-MB-231 only).

MDA-MB-231 cells (left) were treated with MEK inhibitor U0126 (10 μM) for 4 hr, followed by removing the culture-insert, washing and preparing for coculture with L929 cells (right) for time-lapse microscopy. MDA-MB-231 migrated forward in a collective manner, and merged with L929. Each picture frame was taken per 10 minutes. No cell death occurred for both sides. The data is linked to figure S7a and c.

Video S9. Knockout *Wwox*$^{-/-}$ MEF cells dramatically upregulate the redox activity in wild type MEF cells from a remote distance (merged channels).

Wwox wild type and knockout MEF cells were seeded in either side of a culture-insert. Following overnight culture, cells were stained with Redox Sensor Red CC-1 to measure their redox activity, and then subjected to time-lapse microscopy (merged channels from bright field and red fluorescence). When wild type cells detected the presence of migrating knockout cells from a distance of 500 μm (and vice versa), wild type cells rapidly exhibited a significantly upregulated redox activity in less than 2 hr. Note that wild type cells underwent apoptosis in 12 hr under the influence of knockout cells. The original video data are linked to Figure 6a to c.

Video S10. Knockout *Wwox*$^{-/-}$ MEF cells dramatically upregulate the redox activity in wild type MEF cells from a remote distance (red fluorescence only).

The experiment is the same as in Video S9. The red fluorescence is shown only.

Video S11. Wild type versus wild type MEF cells in time-lapse microscopy (merged channels).

The experiment was carried out similarly to that in Videos S9 and S10. When wild type faced wild type cells in either side, little or no induced redox activity and no apoptosis were observed

(merged channels from bright field and red fluorescence). The original video data are linked to Figure 6b and c.

Video S12. Wild type versus wild type MEF cells in time-lapse microscopy (red fluorescence only).
The experiment is the same as in Video S11. The red fluorescence is shown only.

Videos S13. MDA-MB-435s versus wild type MEF cells in time-lapse microscopy.
When WWOX-deficient breast MDA-MB-435s migrated versus wild type MEF cells, apoptosis occurred mainly in the wild type cells.

Videos S14. MDA-MB-231 cells induce a greater extent of L929 apoptosis under serum-free conditions.
Under serum-free conditions, MDA-MB-231 induced a greater numbers of L929 cells undergoing apoptosis. This video data is linked to Figure S10b.

Video S15. Restoration of WWOX in MDA-MB-231 allows them to fend off WWOX-negative parental cells.
MDA-MB-231 cells were treated with methylation inhibitor 5-aza-2' deoxycytidine (5-aza, 5μM) for 5 days to restore WWOX expression (see Fig. 6f). By time-lapse microscopy, parental MDA-MB-231 underwent retrograde migration (right) upon facing 5-aza-treated cells (left) (see blue triangle). This video is linked to Fig. 6e to h.

Video S16. Ectopic expression of the *N*-terminus of WWOX allows MDA-MB-231 to merge with L929.
MDA-MB-231 cells were transiently transfected with an expression construct for EGFP-WW1/2 domains (the *N*-terminal head and the two WW domains). These cells moved forward and then merged with L929. All cells were stained with Cell Tracker Red. This video data is linked to fig. S10d.
